# Effect of Dual-Organic Cations on the Structure and Properties of 2D Hybrid Perovskites as Scintillators

**DOI:** 10.1021/acsami.4c01741

**Published:** 2024-05-03

**Authors:** Md Abdul Kuddus Sheikh, Francesco Maddalena, Dominik Kowal, Michal Makowski, Somnath Mahato, Roman Jȩdrzejewski, Romakanta Bhattarai, Marcin Eugeniusz Witkowski, Konrad Jacek Drozdowski, Winicjusz Drozdowski, Cuong Dang, Trevor David Rhone, Muhammad Danang Birowosuto

**Affiliations:** † Łukasiewicz Research Network-PORT Polish Center for Technology Development, Stabłowicka 147, Wrocław 54-066, Poland; ‡ School of Electrical and Electronic Engineering, Nanyang Technological University, 50 Nanyang Avenue, 639798 Singapore, Singapore; § CINTRA UMI CNRS/NTU/THALES 3288, Research Techno Plaza, 50 Nanyang Drive, Border X Block, Level 6, 637553 Singapore, Singapore; ∥ Department of Physics, Applied Physics, and Astronomy, Rensselaer Polytechnic Institute, Troy, New York 12180, United States; ⊥ Institute of Physics, Faculty of Physics, Astronomy, and Informatics, Nicolaus Copernicus University in Toruń, ul. Grudziądzka 5, 87-100 Toruń, Poland

**Keywords:** hybrid perovskites, dual-organic cation, luminescence, lys, scintillators

## Abstract

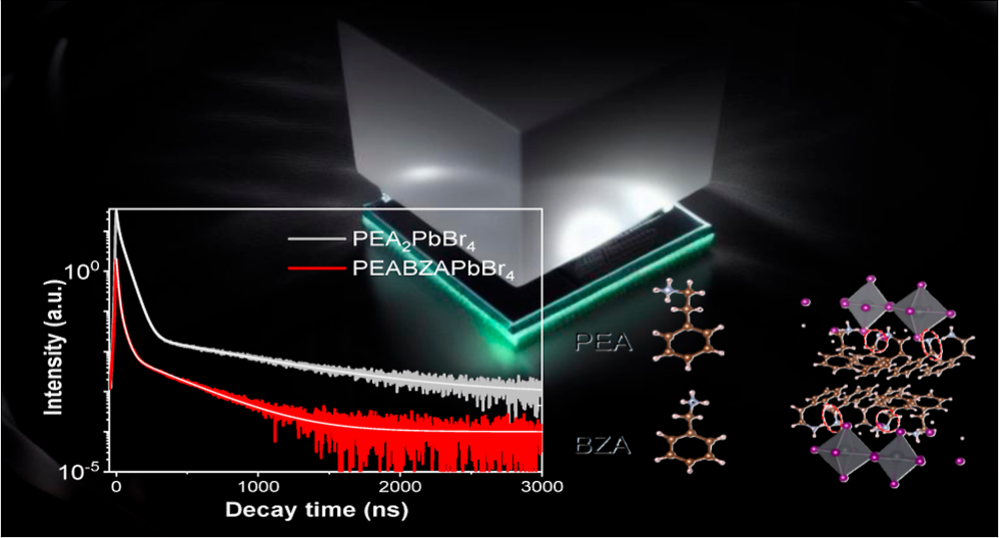

Two-dimensional (2D) hybrid organic–inorganic perovskite (HOIP) crystals show promise as scintillating materials for wide-energy radiation detection, outperforming their three-dimensional counterparts. In this study, we synthesized single crystals of (PEA_2–*x*_BZA_*x*_)PbBr_4_ (*x* ranging from 0.1 to 2), utilizing phenethylammonium (C_6_H_5_CH_2_CH_2_NH_3_^+^) and benzylammonium (C_6_H_5_CH_2_NH_3_^+^) cations. These materials exhibit favorable optical and scintillation properties, rendering them suitable for high light yield (LY) and fast-response scintillators. Our investigation, employing various techniques such as X-ray diffraction (XRD), photoluminescence (PL), time-resolved (TR) PL, Raman spectroscopy, radioluminescence (RL), thermoluminescence (TL), and scintillation measurements, unveiled lattice strain induced by dual-organic cations in powder X-ray diffraction. Density functional theory analysis demonstrated a maximal 0.13 eV increase in the band gap with the addition of BZA cation addition. Notably, the largest Stokes shift of 0.06 eV was observed in (BZA)_2_PbBr_4_. The dual-organic cation crystals displayed >80% fast component scintillation decay time, which is advantageous for the scintillating process. Furthermore, we observed a dual-organic cations-induced enhancement of electron–hole transfer efficiency by up to 60%, with a contribution of >70% to the fast component of scintillation decay. The crystal with the lowest BZA concentration, (PEA_1.9_BZA_0.1_)PbBr_4_, demonstrated the highest LYs of 14.9 ± 1.5 ph/keV at room temperature. Despite a 55–70% decrease in LY for BZA concentrations >5%, simultaneous reductions in scintillation decay time (12–32%) may work for time-of-flight positron emission tomography and photon-counting computed tomography. Our work underscores the crucial role of dual-organic cations in advancing our understanding of 2D-HOIP crystals for materials science and radiation detection applications.

## Introduction

Hybrid organic–inorganic perovskite (HOIP) scintillators with diverse dimensions play a pivotal role in detecting ionizing radiation due to their fast and high light yield, making them particularly well-suited for applications such as imaging, spectroscopy, and timing.^1−5^ Two-dimensional (2D) HOIP crystals are promising scintillating materials for energy detection over a wide range of energies due to their exceptional luminescence efficiency, excellent photoabsorption capability,^6^ high exciton binding energy,^7^ and environmental stability.^8,9^ Particularly noteworthy is the superior tunability and unique optoelectronic properties of 2D-HOIP, which make them highly desirable for scintillation applications.^10,11^ Furthermore, their multiple quantum well-layered structure^12^ facilitates electron confinement effects, thereby limiting electron mobility between organic and inorganic components to a certain extent.^13^ This orderly and controlled process enhances their effectiveness as scintillators for X-ray detection.^14^

Currently, research on phenethylammonium (PEA)-, butylammonium (BA)-, and benzylammonium (BZA)-based lead halide perovskites (A_2_BX_4_; A = PEA, BA, BZA; B = Pb, Sn, Cu, Mn; X = Cl, Br, I)^14^ is of great interest to the scientific community. PEA_2_PbX_4_ crystals have a high light yield (LY) (∼10 photons/keV)^15^ and slow PL decay (∼56 ns).^16^ On the other hand, (BZA)_2_PbX_4_ crystals have a low LY (<6 photons/keV).^13,17^ Furthermore, growing large crystals of (BZA)_2_PbX_4_ is challenging, because of its crystal structure. The PL decay is faster (<3 ns)^13^ compared to PEA or BA cation-based HOIP.^16^ To solve this issue, solvent engineering, halide exchange, and dual-organic cation perovskites are potential candidates. Of all building blocks, dual-cation HOIP single crystals are considered the most optimal platform for fundamental research. Seok et al. prepared iodide-based perovskite thin films to get a tunable band gap and a stable structure by combining the sizable FA cation with the compact MA cation.^18^ Zhu et al. synthesized similar iodide-based perovskite thin films by mixing Cs instead of MA, which provided an improvement in thermal stability.^19^ Recently, Jin et al. published an article in which they utilized mixed organic PEA and inorganic Cs cations, varying the number of layers in lead bromide perovskites and characterized their properties for optoelectronic applications.^20^ Among the numerous reported mixed-organic cation perovskites-based optoelectronic devices, most are based on solar cells.^21−24^ Wu et al. studied the effects of mixed organic cations on the structure and properties of three-dimensional lead halide perovskites.^25^ However, there have been hitherto no reports available on dual-organic cation A_2_PbBr_4_ materials being utilized as scintillators. Furthermore, the precise role of organic cations in perovskites and their impact on material properties remains unclear. To gain a more profound understanding of these material properties, dual-organic cation perovskites would be a suitable candidate.

In this work, we incorporated PEA (C_6_H_5_CH_2_CH_2_NH_3_^+^) and BZA (C_6_H_5_CH_2_NH_3_^+^) to the A site of the A_2_PbBr_4_ perovskite structure to explore the influence of dual-organic cations on structure, optical characteristics, and scintillation properties. The ratio of the cations in the precursor was varied from *x* = 0.1 to *x* = 2.0 in the structure of (PEA_2–*x*_BZA_*x*_)PbBr_4_ to tune the stability of the crystal and optical band gap of the materials. We conducted X-ray diffraction (XRD) experiment for the characterization of the crystal structure, and we confirmed the presence of dual-organic cation inclusion with Raman spectroscopy. We then performed Rietveld refinement of the XRD data and used density functional theory (DFT) to compute the electronic band structure and density of states (DOS). Further on, we observed the effects of dual-organic cations through measurements of absorption and photoluminescence spectra. We also conducted radioluminescence (RL) measurements in a broad range of temperatures to explore the effects of dual-cations on scintillation properties and the afterglow of the perovskite. Also, thermoluminescence (TL) measurements were conducted to study the trap characteristics of 2D-HOIP crystals. Finally, we investigated the effect of dual-cation on the LY of the crystals as we measured the γ-ray pulse height spectra (PHS) with a ^241^Am (*E*_γ_ = 59.5 keV) source. From the obtained results, we observe contraction of the lattice cells for (PEA_1.5_BZA_0.5_)PbBr_4_ and (PEABZA)PbBr_4_ crystals as compared to (PEA)_2_PbBr_4_ crystals. The lattice contraction leads to larger values of the band gaps for the 2D-HOIP crystals with an increase of 0.06 and 0.08 eV for (PEA_1.5_BZA_0.5_)PbBr_4_ and (PEABZA)PbBr_4_, respectively, compared to (PEA)_2_PbBr_4_. Additionally, dual-cation HOIPs also show narrower PL spectra and enhance the efficiency of electron–hole transfer. This study gives better insight on how the dual-cation inclusion results in lattice contraction and narrowing of the band gap, which leads to an increase in scintillation decay time. The comparable LY and faster scintillation decay present dual-cation 2D-HOIP crystals as very promising for improving the scintillation performance of 2D-HOIPs in radiation detection applications.

## Results and Discussion

**Figure 1:**
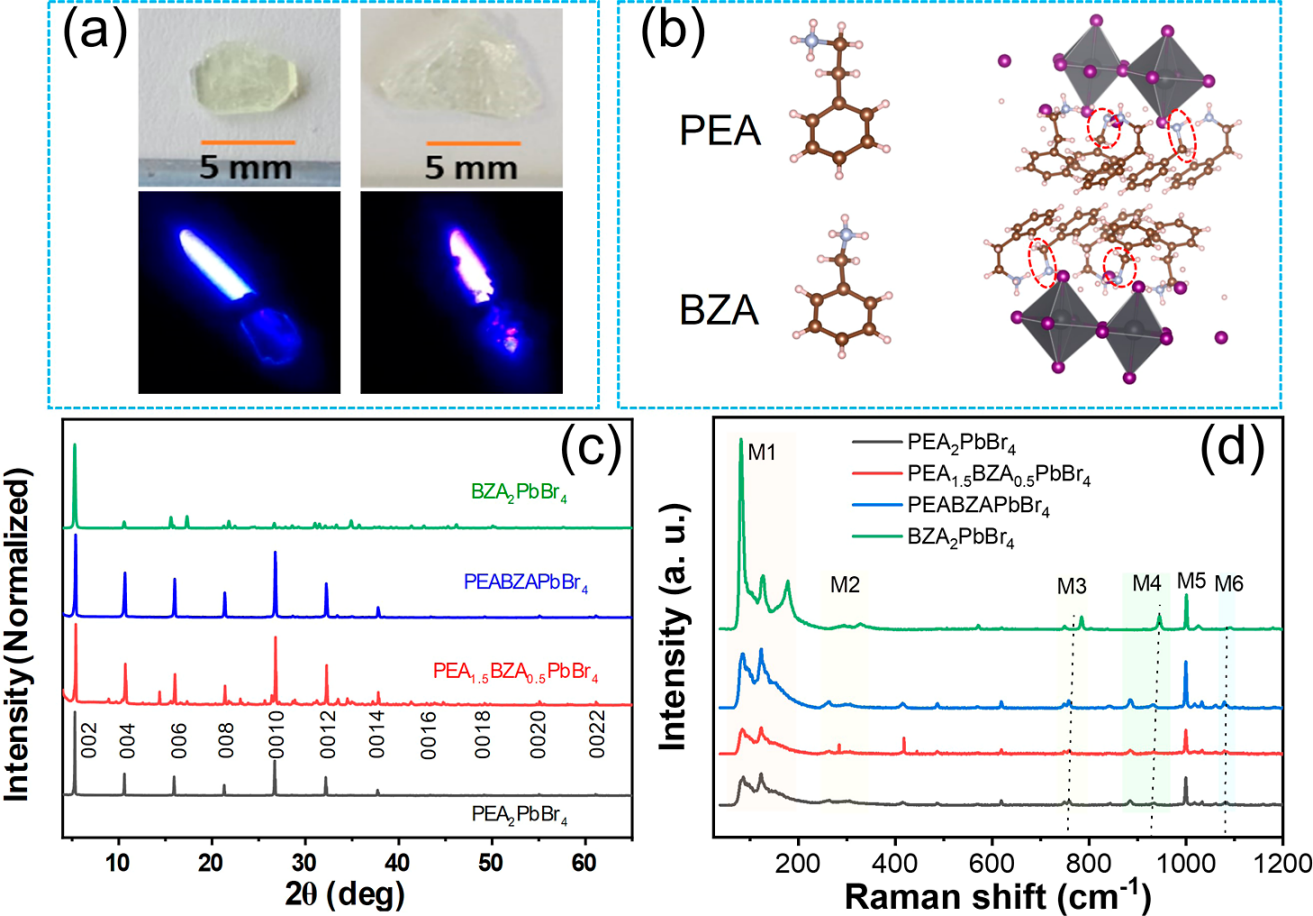
(a) Photographs of (PEA)_2_PbBr_4_ and (PEABZA)PbBr_4_ crystals (top) and blue emission images under 405 nm laser excitation (bottom), (b) chemical structure of PEA (top), BZA (bottom) organic ligands, and crystal structure of (PEABZA)PbBr_4_. The red dotted circle represents the BZA ligand chain, (c) X-ray diffraction pattern of the crystals, and (d) Raman spectra of the samples recorded at room temperature (RT) under laser excitation at 532 nm within the range from 38 to 1200 cm^–1^. The vibrational bands M1–M6 are indicated by the transparent colored rectangles.

Figure 1 depicts the appearance of crystals, blue emission under 405 nm laser excitation, crystal structure, and corresponding XRD pattern. The samples in Figure 1a have an approximate size of 5.0 × 3.7 × 1.0 mm^3^ and 7.8 × 4.5 × 0.3 mm^3^ for (PEA)_2_PbBr_4_ and (PEABZA)PbBr_4_, respectively. The materials comprise colorless platelike crystals that emit blue light when excited with a 405 nm wavelength excitation, as shown in the bottom of Figure 1a. In addition, the emission of the samples is shifted to a shorter wavelength in comparison with (PEA)_2_PbBr_4_. The chemical structures of PEA and BZA are shown in Figure 1b (left), which are widely used for 2D HOIP synthesis. One of the dual-organic cation layered crystal structures of (PEABZA)PbBr_4_ in this study is represented in Figure 1b (right). The PEA ligand is substituted by the BZA ligand in the crystal phase as indicated in the red dotted circle for (PEABZA)PbBr_4_. (PEA)_2_PbBr_4_ crystals contain a total of 12 organic PEA ligands, while (BZA)_2_PbBr_4_ crystals have 8 organic BZA ligands. Upon transformation into (PEA_1.5_BZA_0.5_)PbBr_4_, there are 8 PEA and 4 BZA ligands. The crystal of (PEABZA)PbBr_4_ is formed by combining 6 PEA ligands from (PEA)_2_PbBr_4_ with 4 BZA ligands from (BZA)_2_PbBr_4_. Powder XRD measurements were employed to ascertain the concurrent intercalation of the PEA and BZA cations, as depicted in Figure 1c; their Rietveld refinement with crystal structure using FullProf software was done with the previous models of (PEA)_2_PbBr_4_^16^ and (BZA)_2_PbBr_4_^26^ and the results are shown in Figure S1. Significant peak shifts to higher angles were detected in mixed samples, indicating that incorporating BZA organic cations into the PEA organic cation in the perovskite structure causes structural changes. On the one hand, the triclinic phase can be found with the *P*1® space group for (PEA)_2_PbBr_4_, (PEA_1.5_BZA_0.5_)PbBr_4_, and (PEABZA)PbBr_4_. This phase remains within the broader category of A_2_PbX_4_ (X = I, Br, Cl and A = organic cations, PEA or BZA) HOIP crystals. It comprises a stack of perovskite inorganic layers oriented along the 100 direction, forming a 2D network of Pb-X octahedra,^11^ alternating with the organic sheets of C_6_H_5_(CH_2_)_2_NH_3_^+^ or and C_6_H_5_CH_2_NH_3_^+^ cations. On the other hand, orthorhombic phase with the *Cmc*2_1_ space group^27^ can be found for (BZA)_2_PbBr_4_ and the Rietveld refinement lattice parameters for all samples are summarized in Table S1. In our studies, the lattice parameters of (BZA)_2_PbBr_4_ (*a* = 33.4243 Å, *b* = 8.1558 Å, *c* = 8.1472 Å), the volume of 2220.94 Å^3^, and the calculated density (ρ) of 2.23 g/cm^3^ are similar to the reported lattice parameters (*a* = 33.4062 Å, *b* = 8.1528 Å, *c* = 8.1385 Å), the volume of 2216.63 Å^3^, and the calculated density (ρ) of 2.23 g/cm^3^ in ref (26) (BZA)_2_PbBr_4_ consisted of inorganic layers of corner-sharing PbBr_6_ octahedra, with each separated by two monovalent (BZA) organic cations, as observed in Figure S1 (d). The intense diffraction peaks at 5.3, 10.6, 15.9, 21.3, 26.7, 32.2, and 37.7° were observed, and as the BZA content increased, the diffraction peak shifted toward larger angles, aligning with observations on smaller BZA cations. This shift can be elucidated using the Bragg equation. Given that the size of the BZA cation is smaller than that of the PEA cation, the perovskite lattice size gradually decreases with an increasing BZA proportion. According to the equation 2*d* sin θ = *n*λ, where *d* represents lattice spacing, θ is the diffraction angle, *n* is an integer, and λ is the wavelength, and the diffraction angle increases as the perovskite lattice size decreases.^26^ The experimental results are consistent with theoretical predictions, indicating the formation of a mixed phase of (PEA_2–*x*_BZA_*x*_)PbBr_4_, in which both cations are incorporated within the same lattice framework. This is evidenced by the shift in the diffraction angle; rather than the emergence of two distinct peaks of varying intensities at *x* = 1. At *x* = 0.5, a mixed phase of (PEA_1.5_BZA_0.5_)PbBr_4_ was formed with the addition of several small peaks of different intensities, due to the presence of impurities, including (PEA)_2_PbBr_4_, (PEABZA)PbBr_4_, (BZA)_2_PbBr_4_, and a nonstoichiometric form of (PEA_1.5_BZA_0.5_)PbBr_4_. Raman spectra in Figure 1d show the shifts in the vibrational bands of the PEA structure influenced by the BZA cation. Multiple Raman bands were identified within the range of 38 to 1200 cm^–1^ (0.005–0.148 eV), with characteristics dependent on the type of organic cation, temperature, and the linear polarization direction of the incident light.^28^ At RT, two prominent Raman bands (labeled as M1 and M5) are observable in the spectrum, accompanied by a band appearing as a broad shoulder (M2) and some weak bands at their high-frequency side (M3, M4, and M6). A clear Raman shift was observed in the vibrational band, and such shifts in M3, M4, and M6 bands may indicate that the BZA cation may replace the PEA cation sites in the organic ligand. We also notice that (PEA_1.5_BZA_0.5_)PbBr_4_ exhibits two abnormal peaks at ∼290 and 410 cm^–1^ most likely arising from local structural distortions of the bulk crystal structure, probably occurring at the surface of the crystal, possibly due to the presence of impurities or strain.

**Figure 2:**
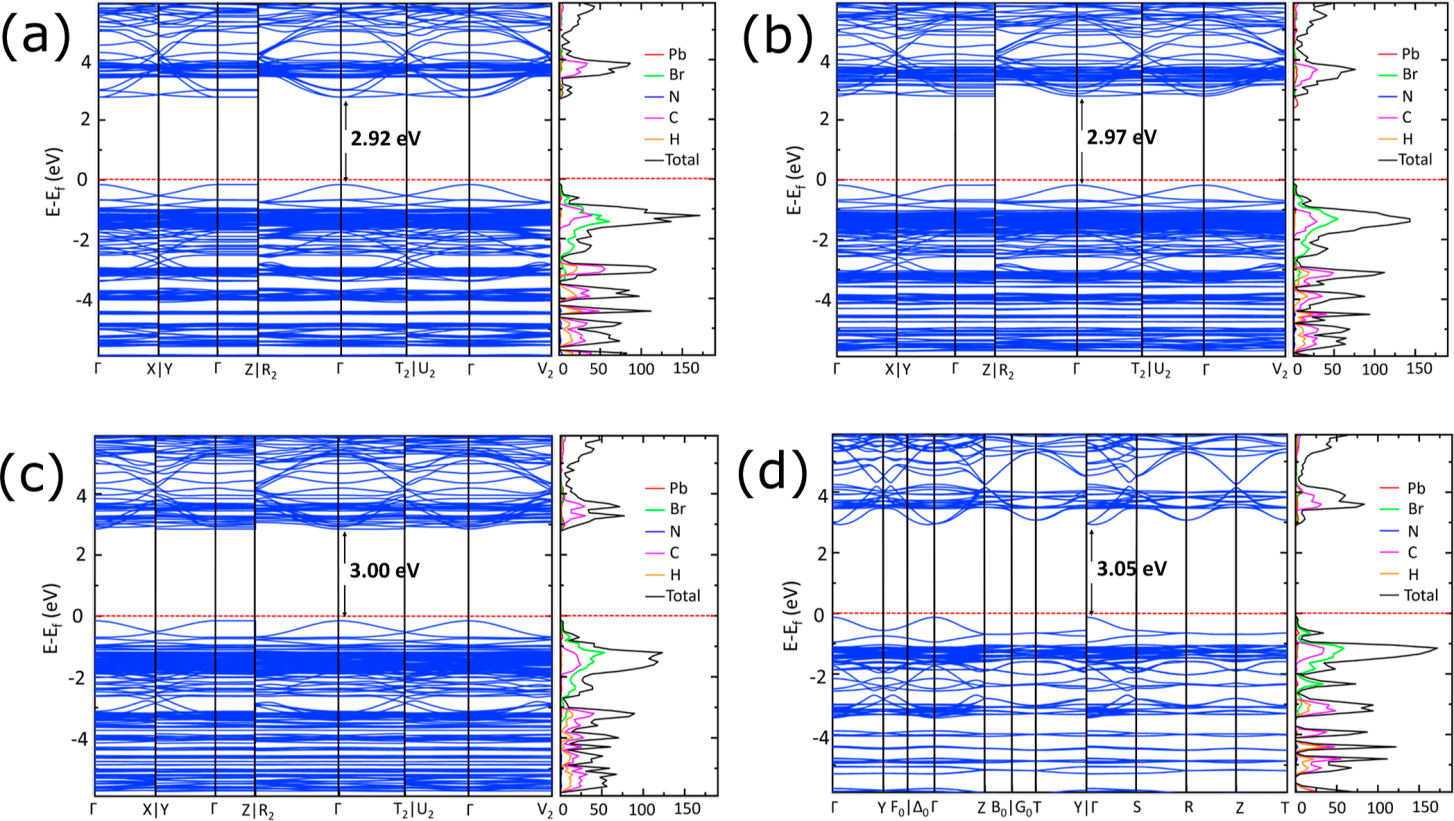
Results of DFT calculations: electronic band structure, total (black), along with projected DOS (indicated by color), are illustrated for (a) (PEA)_2_PbBr_4_, (b) (PEA_1.5_BZA_0.5_)PbBr_4_, (c) (PEABZA)PbBr_4_, and (d) (BZA)_2_PbBr_4_ crystals. In these representations, the Pb p, Br p, N p, C p, and H s orbitals are depicted by red, green, blue, magenta, and orange lines, respectively.

We conducted DFT computations utilizing the cell structure parameters obtained from our XRD measurements outlined in Table S1. The findings of the DFT computations, including both the band structure and the DOS, are depicted in Figure 2. The calculated band gaps (*E*_g_^cal^) are presented in Table S1 for reference. The shift of the band gaps from the calculations for (PEA)_2_PbBr_4_ to (BZA)_2_PbBr_4_ is 0.13 eV and approximately the same with the experimental fitting value of 0.12 eV. Over all, the band gaps obtained from calculations are slightly lower than experimental values, which is reasonable due to self-interaction errors arising from the inherent contribution of density functional parameters.^29,30^ We observe that the lattice contraction and the rise in the band gap provide additional support for the substitution of PEA with BZA in the organic A site. Raman spectroscopy measurements (Figure 1d) additionally indicate a Raman shift in vibrational modes consistent with the substitution of PEA cations with BZA cations in the organic ligand.

**Figure 3:**
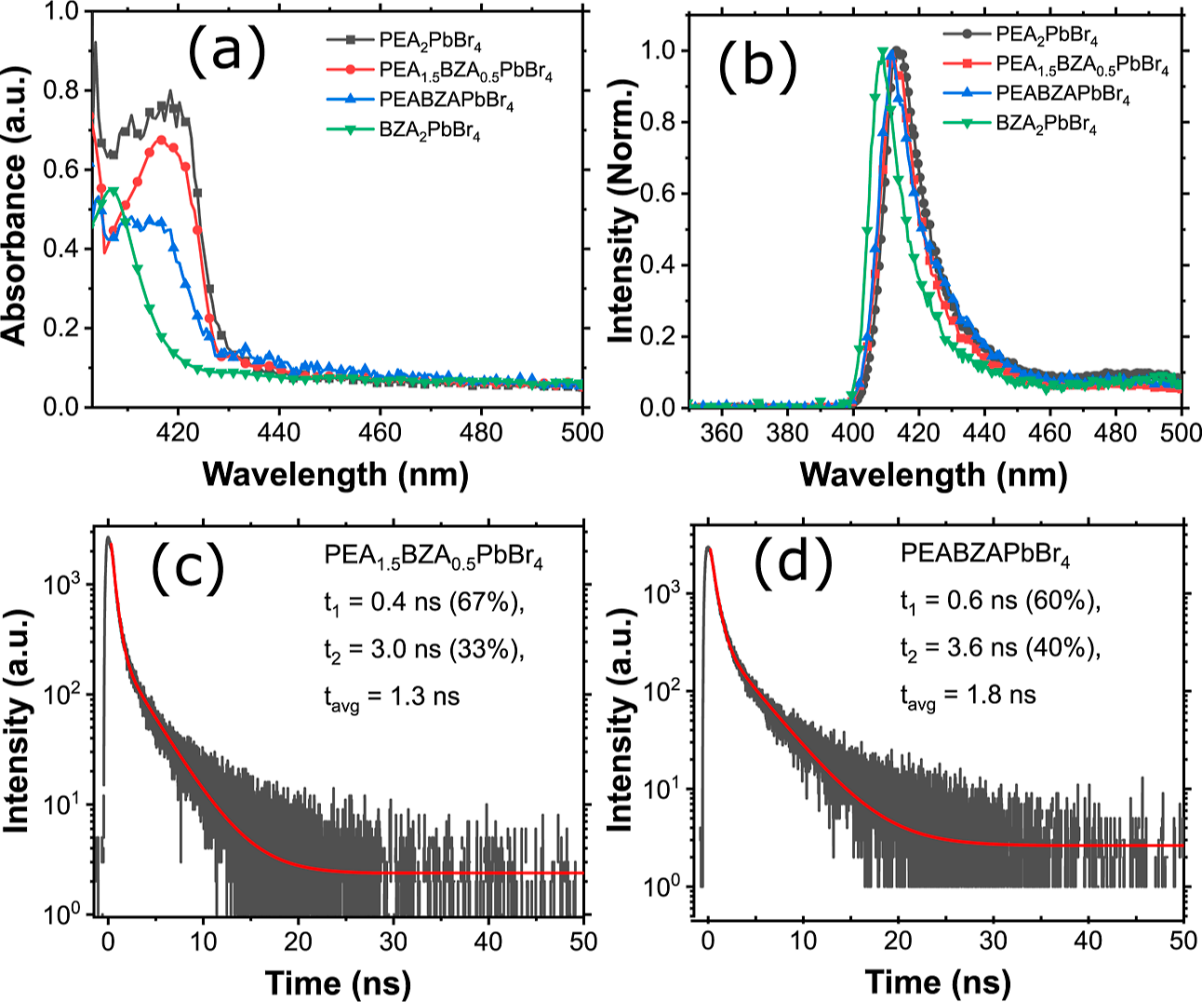
(a) Absorption spectra at RT using white light source, (b) photoluminescence (PL) spectra of (PEA)_2_PbBr_4_, (PEA_1.5_BZA_0.5_)PbBr_4_, (PEABZA)PbBr_4_, and (BZA)_2_PbBr_4_ recorded at RT and using a laser source excited at 375 nm, and TR-PL decay fitting curves (c,d) excited at 375 nm monitoring the ∼412 nm emission of (PEA_1.5_BZA_0.5_)PbBr_4_ and (PEABZA)PbBr_4_, respectively.

We performed UV–vis absorption spectroscopy analysis to ascertain the absorption range and band gap of the dual-cation lead bromide perovskites (PEA_2-x_BZA_*x*_)PbBr_4_, as shown in Figure 3a. The band gap was estimated from the absorption spectra using Elliot’s method for fitting curves, as shown in Figure S2. The experimental band gap from absorption spectra were estimated to be 2.96, 3.02, 3.04, and 3.09 eV for (PEA)_2_PbBr_4_, (PEA_1.5_BZA_0.5_)PbBr_4_, (PEABZA)PbBr_4_, and (BZA)_2_PbBr_4_, respectively, and showed good promise for scintillating materials due to their small values.^31^ By replacing PEA with BZA, we have the capability to fine-tune the band gap of perovskite materials, thereby narrowing the spectral response range while reducing the absorption intensity.

The source of the emitting states in the spectrum was identified through PL and time-resolved PL spectroscopy. PL measurements were performed on bulk crystals with the excitation of a 375 nm laser source and plotted with a logarithmic scale of the *y*-axis recorded at RT, as shown in Figure 3b. The luminescence property of the materials, which is linked with the lattice distortion of the inorganic layer^26^ is characterized using PL measurements. All of the samples displayed blue emission at 412–418 nm wavelengths with an excitation wavelength of 375 nm. A maximum full-width half-maximum (fwhm) value of 14.8 nm (0.26 eV) was observed for (PEA)_2_PbBr_4_ compared to 13.0 nm (0.23 meV), 14.2 nm (0.25 eV), and 11.9 nm (0.21 eV) for (PEA_1.5_BZA_0.5_)PbBr_4_, (PEABZA)PbBr_4_, and (BZA)_2_PbBr_4_ samples, respectively, as shown in Table 1. With the content of BZA increasing, the peak emission shifts toward a shorter wavelength from 418 to 412 nm, due to the lattice contraction, which is consistent with the XRD result. A substantial Stokes shift of 0.06 eV was noted in the case of (BZA)_2_PbBr_4_, whereas a comparatively smaller Stokes shift of 0.01 eV was observed for (PEA_1.5_BZA_0.5_)PbBr_4_. The significant Stokes shift in (BZA)_2_PbBr_4_ can be attributed to its lower degree of self-absorption.^5^ Also due to the experimental configuration with precise excitation beam focusing, the collected emission signal comes in majority from the perovskite crystal surface. Thus we regard the measured PL spectra with only a minor contribution from the self-absorption effect. PL decay curves were fitted with the exponential decay model for (PEA_1.5_BZA_0.5_)PbBr_4_ and (PEABZA)PbBr_4_, as shown in Figure 3c,d. Table S2 displays the calculated average lifetime (*t*_avg_), which considers both time constants and their respective weightings to assess the overall lifetime of the crystals and the parameters derived from the fitting. By the introduction of BZA cation in a pure (PEA)_2_PbBr_4_ perovskite structure, the average decay times for (PEA_1.5_BZA_0.5_)PbBr_4_ and (PEABZA)PbBr_4_ are approximately 4.9 and 3.6 times faster than that for pure (PEA)_2_PbBr_4_,^5^ respectively. Additionally, the (PEA_1.5_BZA_0.5_)PbBr_4_ crystal has a similar decay time (1.3 ns) compared to (PEABZA)PbBr_4_ crystal (1.8 ns) due to the low content of BZA being introduced into both crystal lattices. Moreover, we noted a high percentage of the fast decay component for dual-organic cation crystals compared to single-cation crystals, which is advantageous for the scintillating process as it directly correlates with the recombination rate of excitons within the inorganic layers.^32,33^

**Figure 4:**
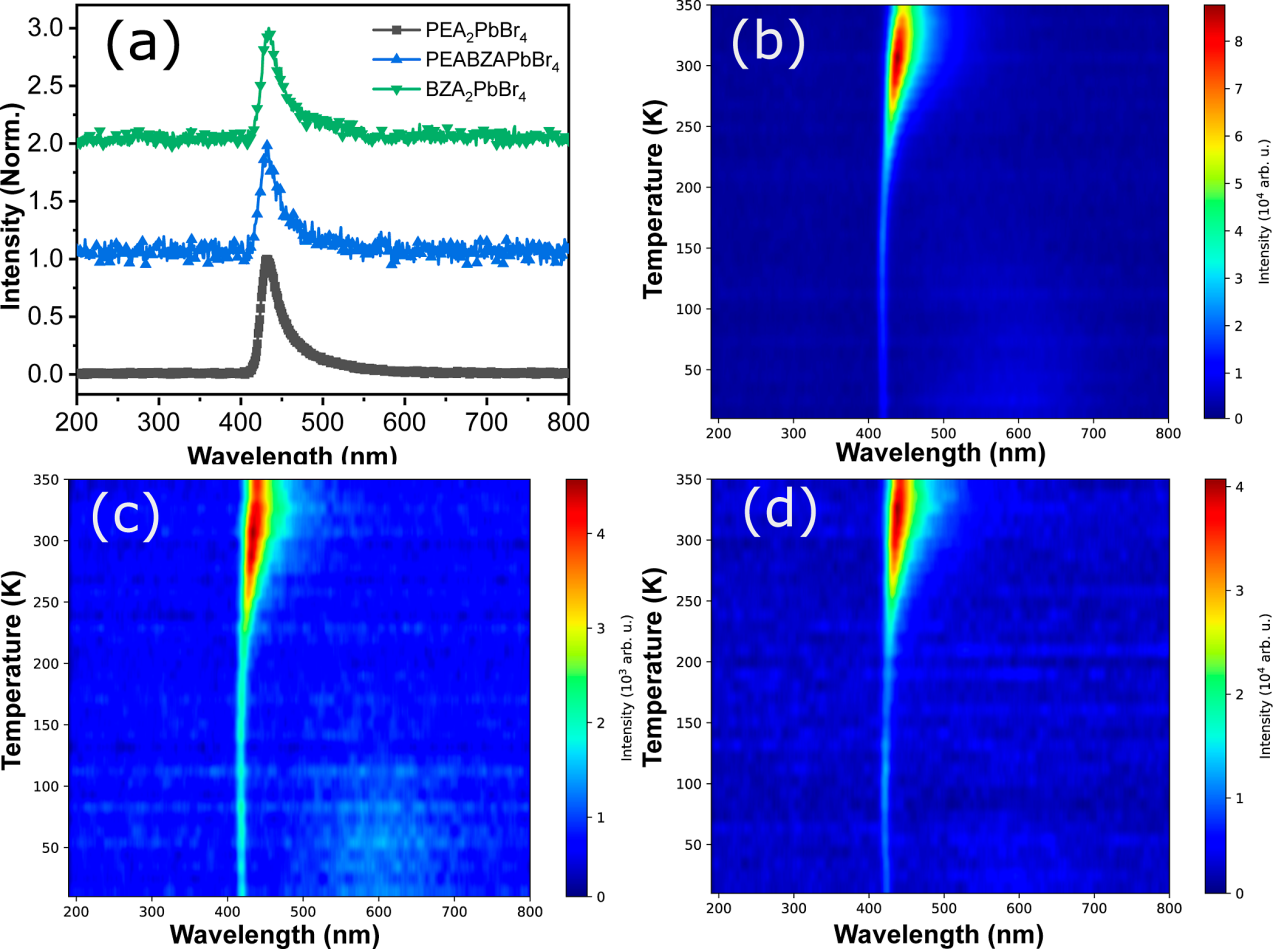
Radioluminescence (RL) spectra (a) at 300 K and mapping spectra at different temperatures from 10 to 350 K for (b) (PEA)_2_PbBr_4_, (c) (PEABZA)PbBr_4_, and (d) (BZA)_2_PbBr_4_ crystals.

RL spectra at 300 K are shown in Figure 4a. A consistent peak was observed across all samples, with variations in intensity among them and both show a fwhm ≈ 28 nm, which is approximately double the PL peaks. There is <4 nm RL peak shift observed with increases in BZA content, while a blue shift was observed in PL emission, as depicted in Figure S3. The intensity of the peaks was normalized to clarify the peak shift. The primary peak signifies the presence of free excitons within the sample, which broadens at high temperatures due to self-absorption.^34−36^ The self-absorption is usually more significant in the case of RL measurements than PL measurements because of the bulk nature of the RL signal.^11^ Because of that the emission peaks are red-shifted by ∼20 nm in the RL spectra as compared to PL. The emission peak linked with free excitation was shifted from 431 to 435 nm as the BZA content was raised. Furthermore, RL spectra mapping of the samples, measured from 10 to 350 K, are illustrated in Figure 4b–d. As the temperature rises, all samples exhibit increased RL intensity, suggesting negative thermal quenching (NTQ) behavior.^37,38^ However, a peak broadening could be observed in (PEA)_2_PbBr_4_, suggesting that some unconventional recombination took place relatively over RT. However, as mixed PEA and BZA were used as organic ligands for the (PEABZA)PbBr_4_ sample, the RL spectra were primarily present at elevated temperatures, resembling RL spectra that were observed for PEA or BZA ligand. This is a remarkable finding since (PEABZA)PbBr_4_ hybrid perovskites that contain both phenethylammonium and benzylammonium groups exhibited maximum peaks at RT.

To investigate the effects of inserting dual-organic cations into the 2D-HOIP structure, we conducted afterglow and TL measurements. We confirmed our findings by using TL measurements, which revealed the presence of trap states within the 2D-HOIP crystals in all samples. The fittings results of the TL curves are displayed in Figure S4, and the parameters of these fittings are displayed in Table S3. (PEA)_2_PbBr_4_ features a trap at the maximum of the peak at 44 K with a trap depth and trap concentration of 0.11 eV and 60 arbitrary units (au), respectively. Several traps at 0.12, 0.14, 0.27, and 0.34 eV are observed for (PEA_1.5_BZA_0.5_)PbBr_4_. However, (PEABZA)PbBr_4_ features less pronounced traps with a small trap width of 0.021 eV and the lowest trap concentration of 30 au compared to the other samples. After exposing our 2D-HOIP crystals to X-rays, we evaluated the residual scintillation response. This exposure occurred in the saturated regime of the scintillation response, and it lasted for 10 min at a temperature of 10 K, aligning with the plateau identified in Figure S5. The parameters governing the exponential decay of the afterglow are detailed in Table S4. With the effect of dual-cations of the 2D-HOIP crystals, the afterglow decay time is observed to increase for both (PEA_1.5_BZA_0.5_)PbBr_4_ and (PEABZA)PbBr_4_. (PEA)_2_PbBr_4_ and (PEABZA)PbBr_4_ feature biexponential decays of the afterglow with average decay times of 22.3 ± 2.2 and 22.8 ± 2.3 s, respectively. (PEA_1.5_BZA_0.5_)PbBr_4_ and (PEA)_2_PbBr_4_, on the other hand, exhibit triexponential decays with average decay times of 58.2 ± 5.8 and 50.1 ± 5.0 s, respectively. (PEA_1.5_BZA_0.5_)PbBr_4_ also shows a prolonged afterglow, with an average fastest decay component lasting for 53.6 ± 5.4 s. Therefore, it is evident that the incorporation of BZA with PEA results in two significant effects: a decrease in decay times and the emergence of a more intricate decay pattern. The augmentation in decay time and the appearance of an extra exponential component can likely be attributed to the introduction of shallow energy traps for the charge carriers, which are induced by the presence of BZA. These shallow traps serve a dual purpose: they either facilitate radiative recombination of the charge carriers or, due to their shallow nature, can be influenced by thermal phonons, potentially leading to charge carrier detrapping. Consequently, this phenomenon contributes to the deceleration of recombination processes within the 2D-HOIP crystals.^16^ This effect is more significant in (PEA_1.5_BZA_0.5_)PbBr_4_, suggesting that BZA introduces more shallow traps.

**Figure 5:**
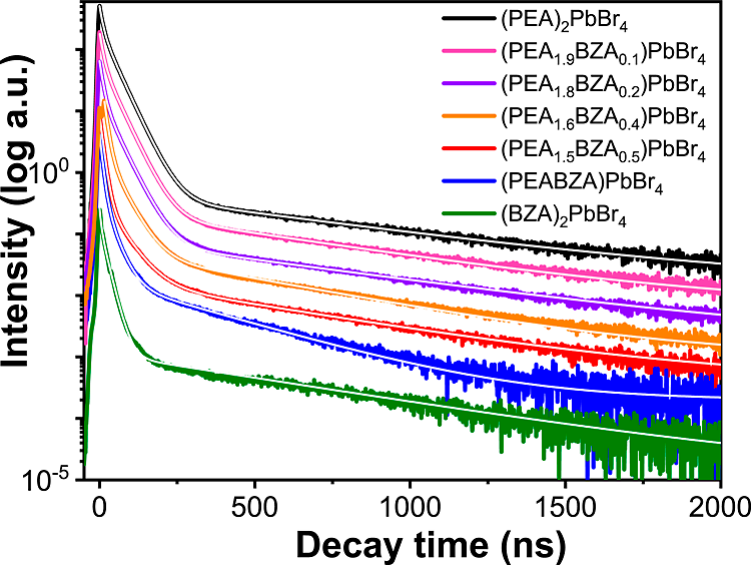
Scintillation decay curves excited by γ-rays at 662 keV and RT with their fitting curves with three exponential decay models for (PEA)_2_PbBr_4_, (PEA_1.5_BZA_0.5_)PbBr_4_, (PEABZA)PbBr_4_, and (BZA)_2_PbBr_4_. The white solid line indicates the fitting of the decay curves.

Figure 5 shows the γ-ray scintillation decay curves of the 2D-HOIP crystals. The fittings of the exponential decay curves are presented in Table S5. Similarly as in the case of PL decay curves, also at high γ-ray energies, the introduction of BZA to PEA results in faster scintillation decay. Both dual-cation samples show faster average decay times compared to single-cation samples. On the other hand, (BZA)_2_PbBr_4_ shows the fastest first component compared to other samples. The average decay times are 43.6 ± 4.4 and 33.4 ± 3.3 ns for (PEA_1.5_BZA_0.5_)PbBr_4_ and (PEABZA)PbBr_4_, respectively. The average decay times for (PEA_1.5_BZA_0.5_)PbBr_4_ and (PEABZA)PbBr_4_ are thus 34 and 49% faster compared to those of (PEA)_2_PbBr_4_ and 12 and 19% faster compared to those of (BZA)_2_PbBr_4_, respectively. The effect of the BZA induces shortening of the scintillation decay times, which is less than the corresponding effect of the TR-PL decay times due to the increase of the trap numbers with high-energy excitation, which is also confirmed by TL measurements, as shown in Figure S4.

**Figure 6:**
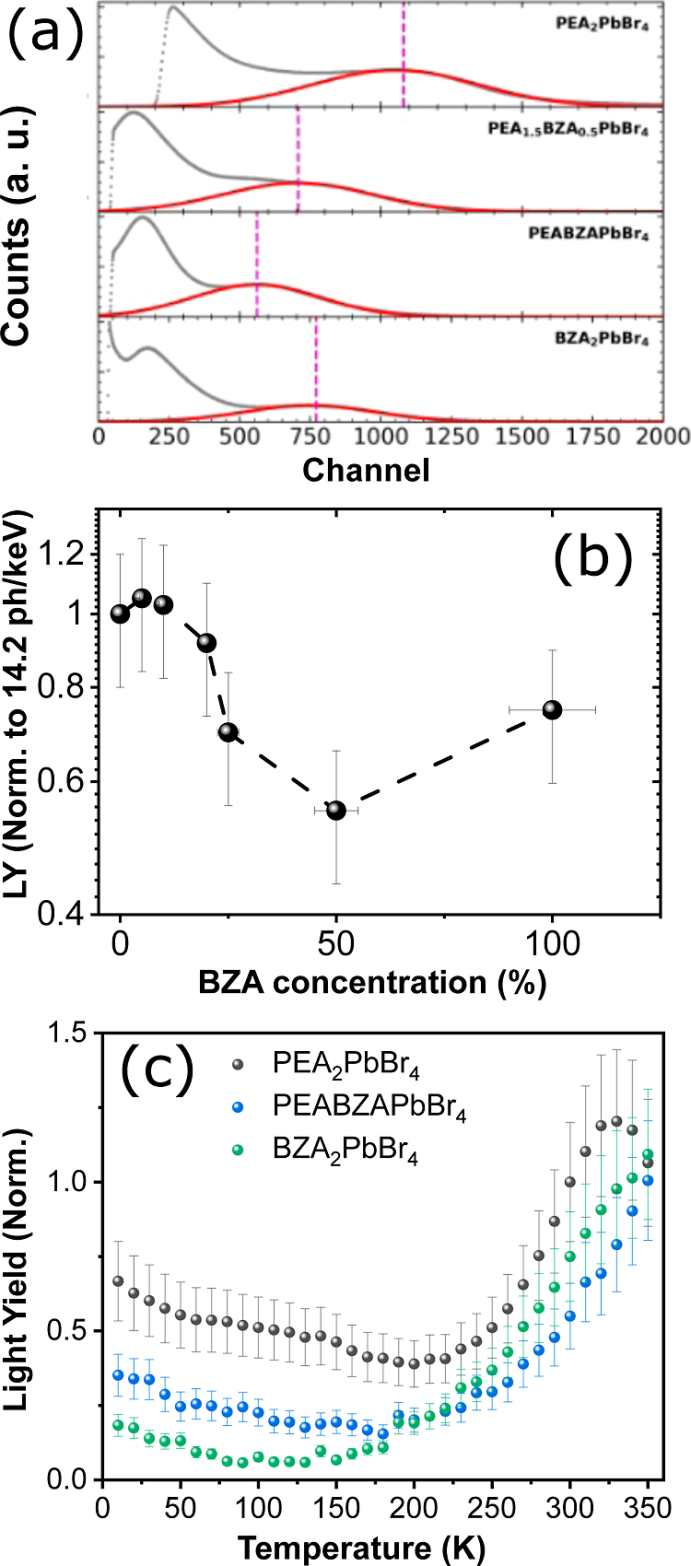
(a) Pulse height spectra (PHS) with 59.5 keV (^241^Am) γ-ray sources for (PEA)_2_PbBr_4_, (PEA_1.5_BZA_0.5_)PbBr_4_, (PEABZA)PbBr_4_, and (BZA)_2_PbBr_4_. The dotted line indicates the positions of the full-energy peak, and the red line shows the related Gaussian fits, (b) BZA concentration vs LY, normalized to the LY of (PEA)_2_PbBr_4_, and (c) temperature-dependent LY changes, normalized to the LY of (PEA)_2_PbBr_4_ at 300 K obtained with PHS and then derived using integrated RL intensities under 45 keV X-ray excitation for (PEA)_2_PbBr_4_, (PEA_1.5_BZA_0.5_)PbBr_4_, (PEABZA)PbBr_4_, and (BZA)_2_PbBr_4_ at 300 K.

Figure 6a shows the pulse height spectra (PHS) for all measured samples using 59.5 keV (^241Am^) γ-ray sources. The LYs of PEA-and BZA-based HOIPs are 14.2 ± 1.4 and 10.6 ± 1.1 ph/keV, respectively; the mix (PEA_2-x_BZA_*x*_)PbBr_4_ (from *x* = 0.1 to *x* = 2) samples have comparatively lower (<10 ph/keV) LYs. The highest LY of 14.9 ± 1.5 ph/keV is observed for 5% BZA concentration, which is 5% higher than that for (PEA)_2_PbBr_4_, and the measured LY values for all samples are summarized in Table S6. The LY gradually decreases from 5% as the BZA concentration increases, and the minimum LY is observed at 50% BZA concentration, as shown in Figure 6b. In this study, the LY of (BZA)_2_PbBr_4_ (10.6 ± 1.1 photons/keV) is approximately 43% larger than in the previous reported value of 6 photons/keV.^13^ The enhancement in LY could be associated with an enhancement in the crystallinity of the (BZA)_2_PbBr_4_ sample, resulting in better resistivity against moisture. An additional PHS measured with 662 keV (^137Cs^) γ-ray source for (BZA)_2_PbBr_4_ sample shows the best energy resolution, as shown in Figure S6.

**Table 1:** Summary of Optical Properties, where *E*_g_^abs^ and *E*_g_^cal^ are the Band Gaps Acquired through Elliot Fitting, as Illustrated in Figure S2, and DFT Calculation, as Depicted in Figure 2, Respectively

compounds	*E*_g_^cal^ (eV)	*E*_g_^abs^ (eV)	maximum PL peak (eV)	Stokes shift (*E*_g_^abs^ ∼ Max.^PL^), (eV)	fwhm (eV)
(PEA)_2_PbBr_4_	2.92	2.96	3.00	0.04	0.26
(PEA_1.5_BZA_0.5_)PbBr_4_	2.97	3.02	3.01	0.01	0.23
(PEABZA)PbBr_4_	3.00	3.04	3.01	0.03	0.25
(BZA)_2_PbBr_4_	3.05	3.09	3.03	0.06	0.21

To discuss in detail the decrease of LY at high BZA concentrations, we relate the LY with the optical properties obtained in the early investigation.^26^ As the band gap increases with BZA content, the LY decreases according to the equation, LY^calc^ ≈ *S*·QY*/E*_g_, where *E*_g_ is the band gap, *S* is the charge transport parameter, QY is the quantum yield derived from the absorbance, refractive index, and PL spectra recorded with the integrating sphere of crystals, as shown in Figure S7.^31,39^ Those parameters and the experimental LY values (LY^exp^) are compiled in Table 2. Yet, in practical applications, the increase in LY may not show linear behavior with the band gap decrease, as cations could influence the quantum yield and charge transport within the material.^26^ For QY, the decrease occurs in tandem with the increase in BZA concentration, and this effect can be anticipated due to the reduction in refractive index;^40^ see Table S7. Using the QYs, it is noted that the highest LY^calc^ value in ph/keV range surpasses its LY^exp^, a pattern consistently observed in other studies on scintillation.^13^ Despite having a relatively lower QY compared to the other samples, the highest *S* was observed at (60.77 ± 6.08). The trend of a more efficient S in contrast to a less efficient QY suggests that defects play a crucial role in inducing transport.^41−43^

**Table 2:** Quantum Yield (QY)^a^ at RT, Calculated LY (LY^calc^), Experimental LY (LY^exp^) Measured at 300 K, and Electron–Hole Transfer Efficiency (*S*)

compounds	QY (%)	LY^calc^ (*S* = 1) (ph/keV)	LY^exp^ (ph/keV)	*S* (%)
(PEA)_2_PbBr_4_	38.2 ± 3.8	56.1 ± 5.6	14.2 ± 1.4	27.51 ± 2.75
(PEA_1.5_BZA_0.5_)PbBr_4_	12.3 ± 1.2	17.7 ± 1.8	9.9 ± 1.0	60.77 ± 6.08
(PEABZA)PbBr_4_	10.7 ± 1.1	15.3 ± 1.5	7.8 ± 0.8	55.09 ± 5.51
(BZA)_2_PbBr_4_	19.3 ± 1.9	27.2 ± 2.7	10.6 ± 1.1	42.29 ± 4.23

Moreover, we measured PHS to obtain the LY^exp^ derived from integrated RL intensities as a function of temperature, as shown in Figure 6c. We chose RT PHS data to assess the temperature dependence of the RL light yield, taking into account considerations of realism, practicality, applicability to diverse fields, and convenience of experimentation. Based on our measurements, we note a slight decline in LY from 10 K, reaching a nadir between 100 and 190 K, followed by an increase, which persists up to the highest temperature recorded. A similar trend is observed for (PEA)_2_PbBr_4_, with a decrease from 10 K to a minimum of about 190 K, then increases, continuing to rise to a maximum at 330 K, and then the LY starts to decrease again. Nevertheless, whereas the majority of materials exhibiting NTQ typically achieve their peak LY within the range of 100–200 K, our measured samples demonstrate their maximum LY at significantly higher temperatures. This characteristic renders this 2D-HOIP device particularly appealing for applications requiring operation at RT and elevated temperatures. We fitted the temperature-dependent LY using an analytical Shibata model^37^ for (PEA)_2_PbBr_4_, (PEABZA)PbBr_4_, and (BZA)_2_PbBr_4_, as shown in Figure S8. The fitting procedures are elaborated upon in the Supporting Information, and the fitting parameters are summarized in Table S8. We note that (PEABZA)PbBr_4_ leads to a decrease in the typical thermal quenching activation energies, as shown in the proposed mechanism energy diagram in Figure S9. In (BZA)_2_PbBr_4_, we observe that the negative thermal quenching activation energies decrease, while the typical thermal quenching activation energy increases significantly. This results in a significant alteration in the NTQ behavior, with the minimum LY shifting toward lower temperatures.

Finally, X-ray imaging measurements were conducted and determined the modulation transfer function (MTF) of the measured samples. Figure S10a shows the photographs of the films prepared for X-ray imaging measurement (details of the preparation of the films are discussed in Supporting Information). Figure S10b shows the X-ray imaging of the best (PEA_1.9_BZA_0.1_)PbBr_4_ sample, and the inset graph represents the normalized counts of Fourier transform of the line spread function vs BZA concentration. The thickness (∼200 μm thick) remains nearly consistent across all samples to mitigate the influence of varying multiple light scattering.^44^ The luminescence (blue emission) was obtained under UV-light for all measured samples, as shown in Figure S10c. To achieve a more objective quantification of the imaging capabilities of our samples, we carried out MTF measurements of our samples, as shown in Figure S10d, and compared them to CsPbBr_3_ nanocrystal and commercial Gadox (Gd_2_O_2_S:Tb) layer samples; see Table S9. There is a similar trend among the light yield, the intensity of the X-ray imaging, and the resolution of all measured samples. The best resolution is obtained from PEA_1.9_BZA_0.1_PbBr_4_, 5.78 lp/mm at 0.2 MTF, which is similar to that of CsPbBr_3_ quantum dots nanocrystals and commercial Gadox samples.^45^ So, our measured samples showcase promising potential for imaging applications, with the MTF closely resembling that of the commercial Gadox scintillator.^45^

## Conclusions

In summary, we have investigated the effect of PEA and BZA cations on the crystal structure and optical and scintillation properties of (PEA_2-x_BZA_*x*_)PbBr_4_ (from *x* = 0.1 to *x* = 2). (BZA)_2_PbBr_4_ exhibits the largest Stokes shift of 0.06 eV among all samples accompanied by a faster PL decay time of 0.9 ns, and a competitive high LY of 10.6 ± 1.1 ph/keV compared to the highest reported value of 6 ph/keV at RT. However, (PEABZA)PbBr_4_ exhibits a fast scintillation decay time of 33.4 ± 3.3 ns, the lowest typical thermal quenching activation energy of >1 meV, which implies that a comparatively lower amount of energy is needed to induce luminescence. We observed a majority of the fast component scintillation decay time (>70%) for dual-organic cation crystals compared to single-cation PEA or BZA crystals (<20%), which is beneficial for the scintillating process. Temperature-dependent RL measurements conducted across all samples indicate a reduction in scintillation emission attributed to thermal quenching. In addition, dual-cation HOIPs enhance the efficiency of electron–hole transfer by up to 60%. The highest LY of 14.9 ± 1.5 ph/keV is observed for 5% BZA concentration, and the minimum LY is observed at 50% BZA concentration. However, such a 5% concentration trend is similarly reported for Li- and Rb-doped (PEA)_2_PbBr_4_ crystals.^16,46^ We can expand this study further into different cations, while the strategy described in this work can be expected to facilitate the advancement of dual-organic-cation HOIP scintillators for radiation detection applications.

## Materials and Methods

### Materials

Dimethyl sulfoxide (DMSO, anhydrous), phenethylammonium bromide ((PEA)Br, ≥98%), benzylammonium bromide ((BZA)Br, ≥98%), and lead bromide (PbBr_2_, ≥98%) were purchased from Sigma-Aldrich. Diethyl ether (99.5%) was purchased from POCH BASIC. All of these chemicals were used without any further purification.

### Synthesis of Crystals

Single or mixed dual-cation crystals of A_2_PbBr_4_ (A = PEA or BZA) were synthesized using a modified version of the previously reported method.^12,46,47^ A precursor solution with 3 M concentration was prepared by dissolving (BZA)Br or (PEA)Br and PbBr_2_ in stoichiometric amounts in DMSO under stirring at 100 °C for 2 h. Crystals were obtained by slowly evaporating DMSO from a 3 M precursor solution in an ambient environment; this process could take a few weeks. Subsequently, the crystal precipitate was washed with diethyl ether and dried under vacuum at 40 °C for 24 h. The obtained perovskite crystals were kept in a glovebox under an inert atmosphere for future characterization.

### X-ray Diffraction

A Bruker D8 Advance AXS diffractometer was used for acquiring the powder X-ray diffraction (XRD) spectra of the synthesized compounds. The device used Cu Kα radiation with a 1.5418 Å wavelength. Measurements were conducted at RT, under Bragg–Brentano geometry, a 5 s/step scanning velocity, and a 0.02° step size. FullProf Suite software was then used to analyze the acquired data. The diffraction patterns were recorded after aligning the crystal in the range of 3 to 65°.

### PL, TRPL, and Absorption

For PL measurements, the samples were excited with the use of a picosecond laser diode with the repetition rate of 30 MHz and a 375 nm wavelength. A microscopic objective facilitated excitation focusing and signal collection. The filtered PL signal was captured by using a high-sensitivity visible light spectrometer. In TRPL measurements, the repetition rate was lowered to 10 MHz, and the emission signal, filtered through a band-pass filter of 532 ± 25 nm, was directed to a single-photon avalanche photodiode (APD). Time-correlated single-photon counting electronics was used to analyze the timing response. A Tungsten Halogen light source (Ocean Optics LS-1) and the same visible light spectrometer as for the PL experiments were used to measure the absorption spectra of the samples in transmission mode. All measurements were conducted at RT, and instruments setup was the same as previously reported.^11^

### RL, TL, and Afterglow Curves

The details of the RL, TL, and afterglow measurements setup and the measurement parameters are reported in a previous study.^11^ TL glow curves were measured from 10 to 350 K by increasing the temperature, with a 0.14 K/s heating rate. Finally, the RL signal was measured from 350 to 10 K by cooling the sample. Measurement started from the highest temperature to avoid the thermal release of charge carriers that could possibly contribute to the emission yield.

### Pulse Height and Scintillation Decay Measurements

The details of the pulse height and scintillation decay measurements were previously reported.^16^ In the pulse height spectrum, the location of the full-energy peak was juxtaposed with the mean value position of the single-electron response to determine the photoelectron yield. The actual LY for the radiation conversion, expressed in photons per MeV, was derived by accounting for the spectral alignment of the sample luminescence with the characteristics of the photomultiplier tube. Scintillation decay measurements were conducted using the delayed coincidence single photon counting method.^10^ No Viscasil grease was used during the measurements to prevent the deterioration of the samples.

### DFT Calculations

The first-principles DFT calculations were conducted using the Vienna ab initio simulation package^48^ that uses the projector augmented wave type of pseudopotential for describing the interaction between valence electrons and ion cores.^49^ We employed the generalized gradient approximation (GGA) based on the Perdew–Burke–Ernzerhof (PBE) functional to account for the exchange–correlation interactions among the electrons.^50^ A Hubbard U parameter of 2 eV was introduced to consider the partially filled p-orbitals of the Pb atoms.^51^ The wave functions were expanded using a plane wave basis set with a cutoff energy of 500 eV. A convergence criterion of 1 × 10^–6^ eV was applied between successive SCF steps for electronic convergence. Full relaxation of the structures is permitted until the force exerted on each atom falls below 0.01 eV/Å between any two successive ionic steps. The Γ-centered *k*-point grids of 1 × 5 × 5 and 3 × 3 × 3 are used for the integration of Brillouin zones for the BA_2_PbBr_4_ and (PEA)_2_PbBr_4_ structures, respectively. The van der Waals corrections are included by the DFT-D3 method.^52^

## Supporting Information Available

Rietveld refinements of crystal XRD, crystal parameters and properties, absorption spectra with Elliot fits, afterglow curves, glow curve fits, pulse height spectra at 662 keV (^137Cs^) γ-ray sources for (BZA)_2_PbBr_4_, PL spectra recorded with integrating sphere of crystals, calculated QY, and scintillation decay parameters at 661.7 keV (^137Cs^) (PDF)
Crystal structure of (PEA)_2_PbBr_4_ (CIF)
Crystal structure of (PEA_1.5_BZA_0.5_)PbBr_4_ (CIF)
Crystal structure of (PEABZA)PbBr_4_ (CIF)
Crystal structure of (BZA)_2_PbBr_4_ (CIF)

## Supplementary Material

am4c01741_si_001.pdf

am4c01741_si_002.cif

am4c01741_si_003.cif

am4c01741_si_004.cif

am4c01741_si_005.cif

## Abbreviations

HOIP: hybrid organic–inorganic perovskites
2D: two-dimensional
PEA: phenethylamine
BZA: benzylamine
RT: room temperature
XRD: X-ray diffraction
DOS: density of states
DFT: density functional theory
PL: photoluminescence
TRPL: time-resolved PL
RL: radioluminescence
NTQ: negative thermal quenching
TL: thermoluminescence
