# Supplementary material for: Effect of Dual-Organic Cations on the Structure and Properties of 2D Hybrid Perovskites as Scintillators
Source: ACS Appl Mater Interfaces. 2024 May 3;16(19):25529–39. doi: 10.1021/acsami.4c01741 (PMC11103655; doi:10.1021/acsami.4c01741)
Supplement: Supplementary file 1 — am4c01741_si_001.pdf [file am4c01741_si_001.pdf]

Supporting Information

Effect of Dual-Organic Cations on the Structure and Properties of 2D Hybrid Perovskites as Scintillators

Md Abdul Kuddus Sheikh[1,*], Francesco Maddalena[2,*], Dominik Kowal[1], Michal Makowski[1], Somnath Mahato[1], Roman Jędrzejewski[1], Romakanta Bhattarai[3], Marcin Eugeniusz Witkowski[4], Konrad Jacek Drozdowski[4], Winicjusz Drozdowski[4], Cuong Dang[2], Trevor David Rhone[3], Muhammad Danang Birowosuto[1,*]

[1] Łukasiewicz Research Network-PORT Polish Center for Technology Development, Stabłowicka 147, Wrocław, 54-066, Poland;

[2] School of Electrical and Electronic Engineering, Nanyang Technological University, 50 Nanyang Avenue, 639798, Singapore; CINTRA UMI CNRS/NTU/THALES 3288, Research Techno Plaza, 50 Nanyang Drive, Border X Block, Level 6, 637553, Singapore;

[3] Department of Physics, Applied Physics, and Astronomy, Rensselaer Polytechnic Institute, Troy, NY, 12180, USA;

[4] Institute of Physics, Faculty of Physics, Astronomy, and Informatics, Nicolaus Copernicus University in Toruń, ul. Grudziądzka 5, 87-100 Toruń, Poland;

* Corresponding author:

Email addresses:

abdul.kuddus-sheikh@ port.lukasiewicz.gov.pl (Md Abdul Kuddus Sheikh),

francesco_maddalena@ntu.edu.sg (Francesco Maddalena),

muhammad.birowosuto@port.lukasiewicz.gov.pl (Muhammad Danang Birowosuto)

List of Figures

Figure S1. Rietveld refinements of single-crystal X-ray diffraction (XRD) spectra (right) and crystal structure (left) of from (a) $(PEA)_2PbBr_4$ (b) $(PEA_{1.5}BZA_{0.5})PbBr_4$, (c) $(PEABZA)PbBr_4$, and (d) $(BZA)_2PbBr_4$ using reference structures from.[1,2] The lattice parameters are shown in Table S1.

Figure S2. Absorption spectra from (a) $(PEA)_2PbBr_4$ (b) $(PEA_{1.5}BZA_{0.5})PbBr_4$, (c) $(PEABZA)PbBr_4$, and (d) $(BZA)_2PbBr_4$ and their fitting curves with Elliot method in Equations S1 and S2.

Figure S3. PL (solid lines) and RL (dotted lines) spectra for $(PEA)_2PbBr_4$ (black), $(PEABZA)PbBr_4$ (blue), and $(BZA)_2PbBr_4$ (Dark Cyan).

Figure S4. The fit of glow curves of (a) $(PEA)_2PbBr_4$, (b) $(PEA_{1.5}BZA_{0.5})PbBr_4$, (c) $(PEABZA)PbBr_4$, and (d) $(BZA)_2PbBr_4$ with multiple Randal-Wilkins method in Eq. S3. [3, 4] The parameters of the fit are shown in Table S3.

Figure S5. Afterglow decay curves at 10 K. The white dotted line show the exponential decay fits.

Figure S6. Pulse height spectra (PHS) with 662 keV (^{137}Cs) γ-ray sources for $(BZA)_2PbBr_4$. The dotted gray line indicate the positions of the escape peak and the full-energy peak the red dotted line shows the sum of both peaks.

Figure S7. PL spectra recorded with integrating sphere of crystals. $(PEA)_2PbBr_4$, $(PEA_{1.5}BZA_{0.5})PbBr_4$, $(PEABZA)PbBr_4$, and $(BZA)_2PbBr_4$ represent black, red, blue, and dark cyan color, respectively.

Figure S8. Fitting parameters of negative thermal quenching of the radioluminescence using equation S5.

Figure S9. An energy diagram of the model system proposed for the study mechanism of the negative thermal quenching phenomenon for (a) $(PEA)_2PbBr_4$, (b) $(PEABZA)PbBr_4$, (c) $(BZA)_2PbBr_4$.

Figure S10. (a) Photographs of the films prepared for X-ray imaging, (b) X-ray imaging of the best $(PEA_{1.9}BZA_{0.1})PbBr_4$ sample (scale bar is 1 mm). The inset shows BZA concentration vs normalized counts, (c) Luminescence (Blue emission) of the samples under UV-light, and (d) Modulation Transfer Function (MTF) of X-ray images obtained from $(PEA)_2PbBr_4$, $(PEA_{1.9}BZA_{0.1})PbBr_4$, $(PEA_{1.5}BZA_{0.5})PbBr_4$, $(PEABZA)PbBr_4$, and $(BZA)_2PbBr_4$ samples with spatial resolution (lp/mm) at 0.2 MTF.

List of Tables

Table S1. Crystal data and structure refinement for $(PEA)_2PbBr_4$, $(PEA_{1.5}BZA_{0.5})PbBr_4$, $(PEABZA)PbBr_4$, and $(BZA)_2PbBr_4$.

Table S2. Parameters of the PL decay curves, where τ_i, C_i and $\bar{\tau}$ represent the decay time, contribution of the decay time and mean time of the decay respectively.

Table S3. Parameters of the thermoluminescence (TL) peak fitting, where T_{max}, E, n_0, and σ represent the temperature where the maximum of the peak occurs, trap depth, trap concentration, and the frequency factor respectively.

Table S4. Afterglow decay parameters, where τ_i, C_i and $\bar{\tau}$ represent the decay time, contribution of the decay time and mean time of the decay respectively.

Table S5. Parameters of the scintillation decay curves, where τ_i, C_i and $\bar{\tau}$ represent the decay time, contribution of the decay time and mean time of the decay respectively. Decay curves were fitting with three exponential decay model.

Table S6. Light Yield obtained from PHS with 59.5 keV (^{241}Am) γ-ray sources at 300 K.

Table S7. Calculated quantum yield (QY) for $(PEA)_2PbBr_4$, $(PEA_{1.5}BZA_{0.5})PbBr_4$, $(PEABZA)PbBr_4$, and $(BZA)_2PbBr_4$ samples.

Table S8. Parameters of the Shibata model for light yield vs temperature, where D_i and C_i corresponds to the amplitudes of Boltzmann distribution, E_{ni} corresponds to the activation energy of each of the intermediate states, and E_{di} corresponds to the activation recombination energy of nonradiative processes.

Table S9. Spatial resolution (lp/mm) at 0.2 MTF for $(PEA)_2PbBr_4$, $(PEA_{1.9}BZA_{0.1})PbBr_4$, $(PEA_{1.5}BZA_{0.5})PbBr_4$, $(PEABZA)PbBr_4$, and $(BZA)_2PbBr_4$, Lamellar with 50% $CsPbBr_3$ nanocrystals and commercial Gadox (Gd_2O_2S:Tb) layer sample.

1. Rietveld refinements and crystal lattice parameters

The X-ray diffraction (XRD) pattern in Figure S1 was analyzed using the Rietveld program Fullprof.[5] The profile function of a Thompson-Cox-Hastings pseudo-Voigt function was used to analyze using the inputs from previous lattice parameters of $(PEA)_2PbBr_4$[1] and $(BZA)_2PbBr_4$.[2] The refinement parameters are summarized in **Table S1**.

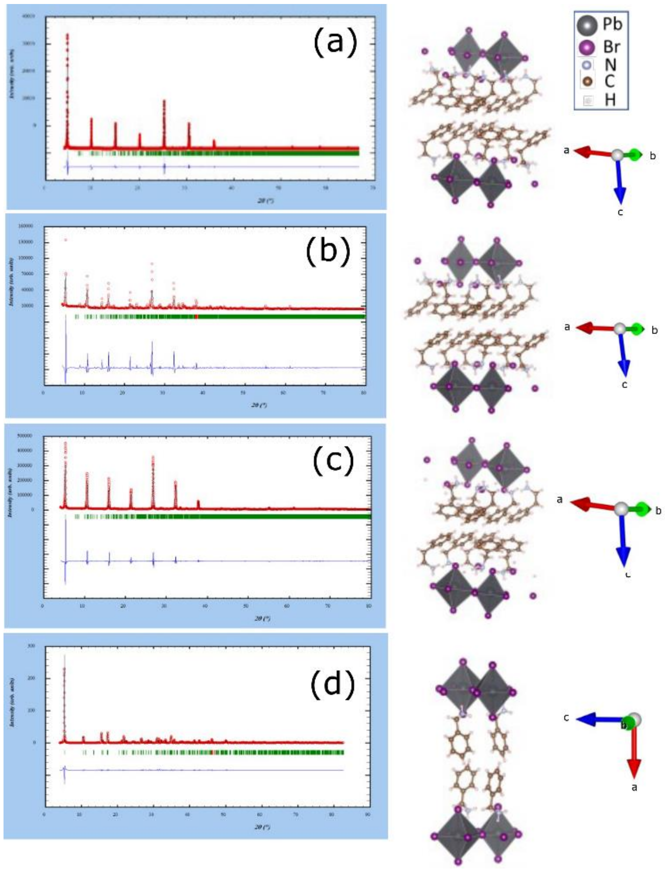

Figure S1. Rietveld refinement of crystal XRD diffractograms of from (a) $(PEA)_2PbBr_4$ (b) $(PEA_{1.5}BZA_{0.5})PbBr_4$, (c) $(PEABZA)PbBr_4$, and (d) $(BZA)_2PbBr_4$ using reference structures from.[1, 2] The lattice parameters are shown in **Table S1**.

Table S1. Crystal data and structure refinement for $(PEA)_2PbBr_4$, $(PEA_{1.5}BZA_{0.5})PbBr_4$, $(PEABZA)PbBr_4$, and $(BZA)_2PbBr_4$.

	$(PEA)_2PbBr_4$	$(PEA_{1.5}BZA_{0.5})PbBr_4$	$(PEABZA)PbBr_4$	$(BZA)_2PbBr_4$
Empirical formula	$C_{16}H_{24}N_2PbBr_4$	$C_{31}H_{46}N_4Pb_2Br_8$	$C_{15}H_{22}N_2PbBr_4$	$C_{14}H_{20}N_2PbBr_4$
Formula weight	771.20	763.95	757.17	743.15
Crystal system	Triclinic	Triclinic	Triclinic	Orthorhombic
Space group	$P\bar{1}$	$P\bar{1}$	$P\bar{1}$	$Cmc2_1$
a (Å)	11.6066	11.5323	11.5739	33.4243
b (Å)	11.6020	11.6731	11.6546	8.1558
c (Å)	17.5882	17.5264	17.5116	8.1472
α (˚)	99.7730	99.6547	99.2473	90.0000
β (˚)	105.4900	105.2685	105.4984	90.0000
γ (˚)	90.0480	90.6438	90.5111	90.0000
V ($Å^3$)	2246.61	2240.03	2243.31	2220.94
Z	4	4	4	4
Calculated density	2.28 g/cm^3	2.26 g/cm^3	2.25 g/cm^3	2.23 g/cm^3

2. Absorption spectra fitting

The fit to absorption spectrum in **Figure S2** was performed by using Elliot formalism.[6] In principle, the contributions to the absorption coefficient (α) can be defined from free carriers (continuum) (α_c) and excitons (α_{ex}).

$$\alpha(\hbar\omega) = \alpha_c + \alpha_{ex} \qquad \text{(S1)}$$

$$\alpha(\hbar\omega) = P_{cv}\left[\,\theta(\hbar\omega - E_g)\cdot\left(\frac{\pi e^{\pi x}}{\sinh(\pi x)}\right) + R_{ex}\sum_{n=1}^{\infty}\frac{4\pi}{n^3}\cdot\delta\left(\hbar\omega - E_g + \frac{R_{ex}}{n^2}\right)\right] \qquad \text{(S2)}$$

where P_{cv} is the frequency dependence and related to the interband transition matrix element, $\hbar\omega$ is the photon energy, $\theta(\hbar\omega - E_g)$ is the heavy side step function, x is defined as p $R_{ex}/(\hbar\omega - E_g)$, and δ denotes a delta function, R_{ex} is exciton Rydberg energy, n is the principle quantum number. From the fits, obtained E_g^{abs} are summarized in **Table S1**.

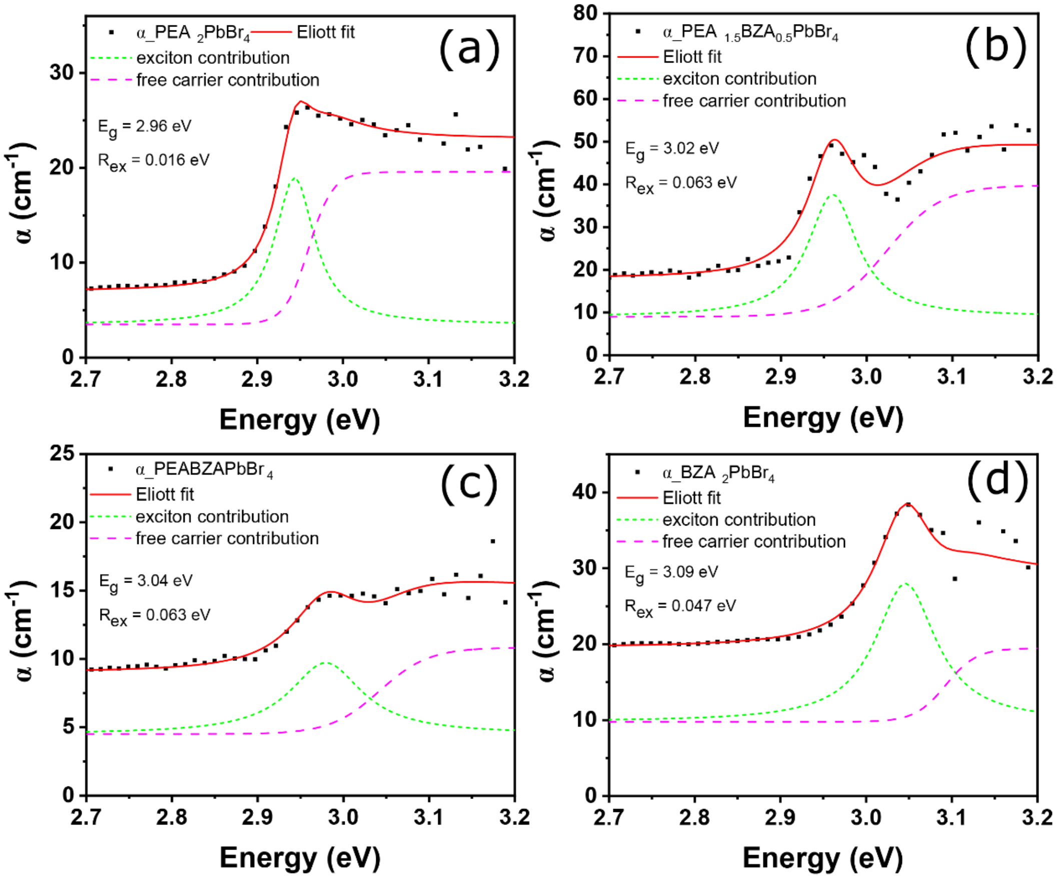

Figure S2. Absorption spectra from (a) $(PEA)_2PbBr_4$ (b) $(PEA_{1.5}BZA_{0.5})PbBr_4$, (c) $(PEABZA)PbBr_4$, and (d) $(BZA)_2PbBr_4$ and their fitting curves with Elliot method in Equations (Eq.) S1 and S2.

Table S2. Parameters of the PL decay curves, where τ_i, C_i and $\bar{\tau}$ represent the decay time, contribution of the decay time and mean time of the decay respectively.

**Compounds**	τ_1 (ns)	C_1 (%)	τ_2 (ns)	C_2 (%)	τ_3 (ns)	C_3 (%)	$\bar{\tau}$ (ns)
$(PEA)_2PbBr_4$[1]	5.2	46	34.6	33	289.7	21	74.6
$(PEA_{1.5}BZA_{0.5})PbBr_4$	0.4	67	3.0	33	-	-	1.3
$(PEABZA)PbBr_4$	0.6	60	3.6	40	-	-	1.8
$(BZA)_2PbBr_4$	0.4	58	3.1	42	-	-	0.9

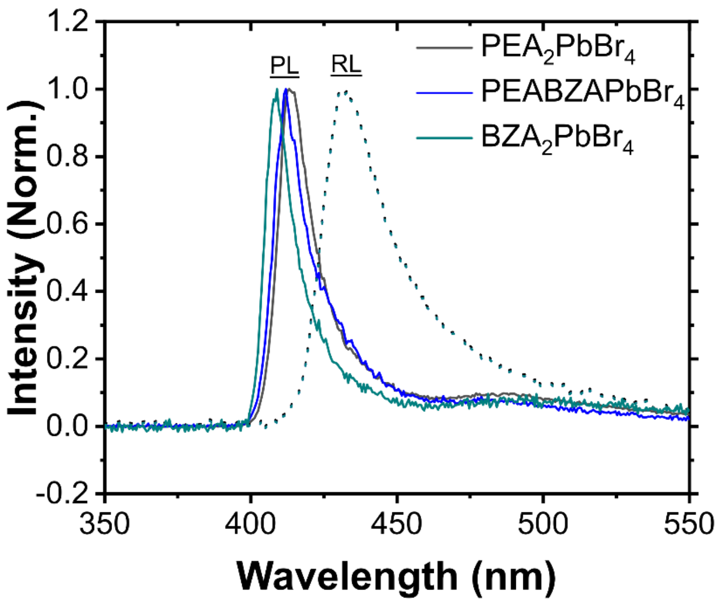

Figure S3. PL (solid lines) and RL (dotted lines) spectra for $(PEA)_2PbBr_4$ (black), $(PEABZA)PbBr_4$ (blue), and $(BZA)_2PbBr_4$ (dark cyan).

3. Glow curves, afterglow, and scintillation decay spectra

Auvoris and Morgan[7] proposed that the broad bands observed in glow curves originate from quasi-continuous Gaussian distributions of traps, rather than arising from a sum of discrete traps (as expressed in equation S3). This method was successfully introduced by Brylew,[8] Drozdowski,[9, 10] and Makowski.[11] Given the reasonable results obtained in terms of trap parameters and fitting accuracy by these previous approaches, we opted to employ this model as well.

To obtain all the necessary parameters, our initial step involved deconvolving the glow curves into peaks using the classic Randall-Wilkins equation. In this equation, A_i and $E0_i$ is an amplitude and energy center of a i-th gaussian envelope, respectively. Parameter s corresponds to the frequency factor, E_j is an j-th energy from gaussian distribution, σ_i is a width of i-th gaussian distribution,β is a heating rate and T is a temperature.

$$I(T) = \sum_i A_i \cdot s \cdot \sum_j exp\left[-\left(\frac{E_j - E_{0_i}}{\sigma_i}\right)^2\right] \cdot exp\left[-\frac{E_j}{kT}\right] \cdot exp\left[-\frac{s}{\beta}\int_{T_0}^{T} exp\left(-\frac{E_j}{k\theta}\right) d\theta\right] \quad \text{(S3)}$$

where T is the temperature, β is the heating rate, k_B is the Boltzmann constant, n_{0i} is the initial trap concentration, V is the crystal volume, E_i is the trap depth, and σ_i is the frequency factor of each

component. The unit-less $n_{0i}V$ or A_i is used to compare afterglow of different crystals. From exponential fits of Eq. S3 to Supplementary Fig. S4, we obtain the parameters and summarized in **Table S3**.

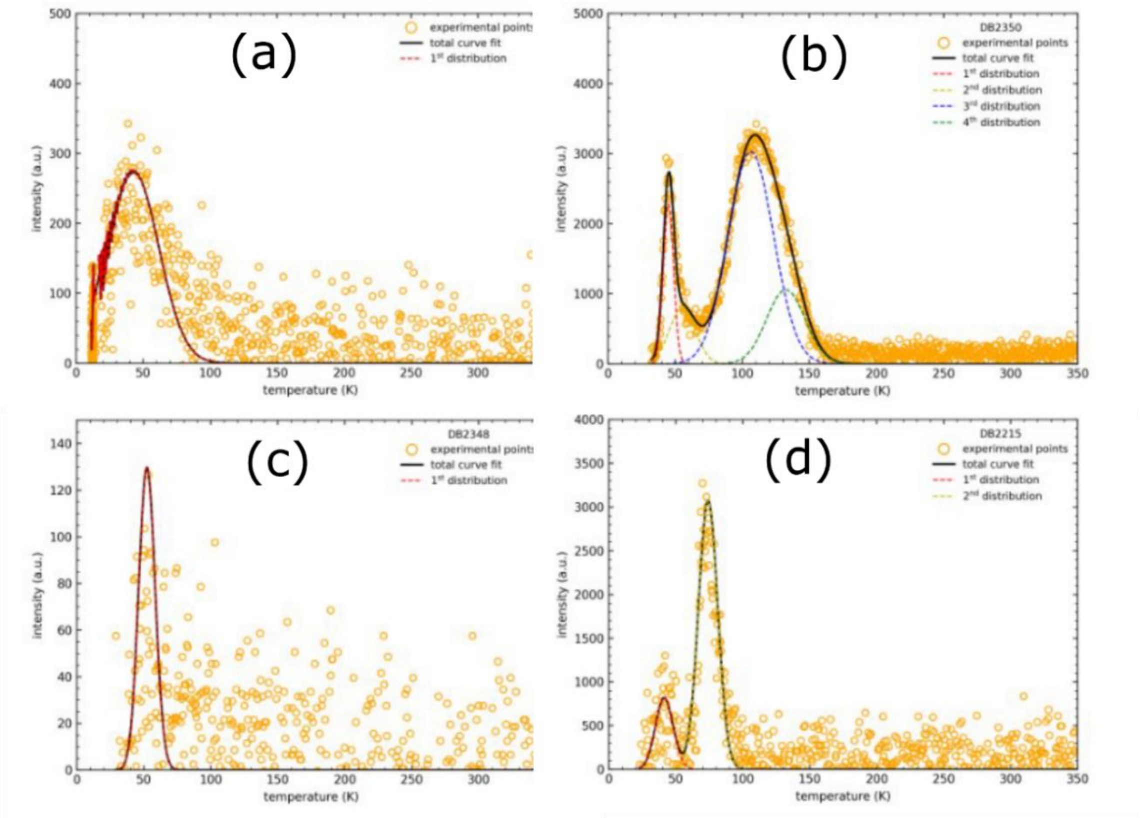

Figure S4. The fit of glow curves of (a) $(PEA)_2PbBr_4$, (b) $(PEA_{1.5}BZA_{0.5})PbBr_4$, (c) $(PEABZA)PbBr_4$, and (d) $(BZA)_2PbBr_4$ with multiple Randal-Wilkins method in Eq. S3.[3, 4] The parameters of the fittings are shown in **Table S3**.

Table S3. Parameters of the thermoluminescence (TL) peak fitting, where T_{max}, E, n_0, and σ_i represent the temperature where the maximum of the peak occurs, trap depth, trap concentration, and the width of i-th Gaussian distribution, respectively.

**Compounds**	T_{max} (K)	E (meV)	n_0 (a.u.)	σ (meV)
$(PEA)_2PbBr_4$	44	105	60	69
$(PEA_{1.5}BZA_{0.5})PbBr_4$	45	117	565	12
	54	138	176	34
	108	270	662	60
	132	339	239	50
$(PEABZA)PbBr_4$	52	130	30	21
$(BZA)_2PbBr_4$	42	102	185	23
	74	186	724	24

The afterglow curves in **Figure 3a** were fitted with exponential decay model. The parameters are shown in **Table S4**.

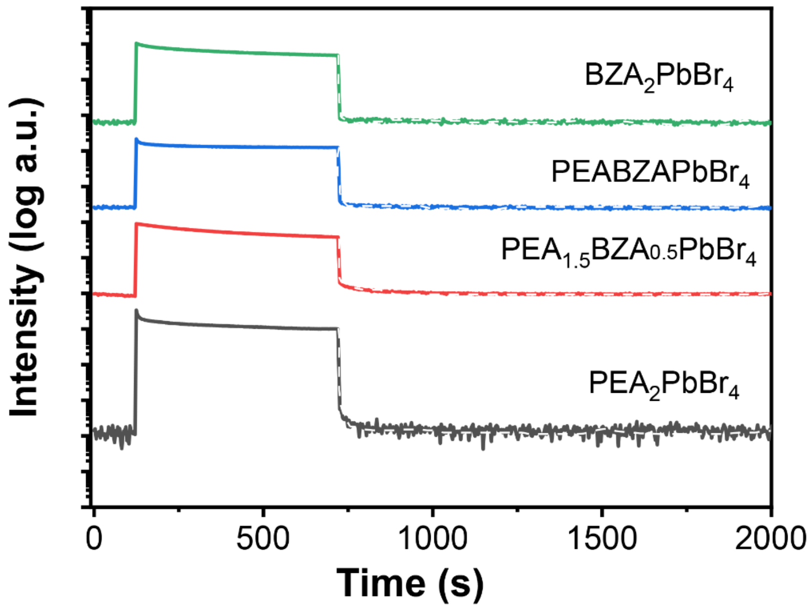

Figure S5. Afterglow decay curves at 10 K. The white dotted line show the exponential decay fits.

Table S4. Afterglow decay parameters, where τ_i, C_i and $\bar{\tau}$ represent the decay time, contribution of the decay time and mean time of the decay respectively.

**Compounds**	τ_1 (s)	C_1 (%)	τ_2 (s)	C_2 (%)	τ_3 (s)	C_3 (%)	$\bar{\tau}$ (s)
$(PEA)_2PbBr_4$	10.3 ± 1.0	83 ± 8	82.2 ± 8.2	17 ± 2	-	-	22.3 ± 2.2
$(PEA_{1.5}BZA_{0.5})PbBr_4$	53.6 ± 5.4	55 ± 5	23.6 ± 2.4	10 ± 1	75.8 ± 7.6	35 ± 3	58.2 ± 5.8
$(PEABZA)PbBr_4$	21.5 ± 2.1	35 ± 3	23.6 ± 2.4	65 ± 6	-	-	22.8 ± 2.3
$(BZA)_2PbBr_4$	17.1 ± 1.7	47 ± 5	10.6 ± 1.1	12 ± 1	98.5 ± 9.8	41 ± 4	50.1 ± 5.0

Scintillation decay curves in **Figure 5** were fitted with three exponential decay model and the parameters are shown in **Table S5**.

Table S5. Parameters of the scintillation decay curves, where τ_i, C_i and $\bar{\tau}$ represent the decay time, contribution of the decay time and mean time of the decay respectively. Decay curves were fitting with three exponential decay model.

**Compounds**	τ_1 (ns)	C_1 (%)	τ_2 (ns)	C_2 (%)	τ_3 (ns)	C_3 (%)	$\bar{\tau}$ (ns)
$(PEA)_2PbBr_4$	9.5 ± 0.9	20 ± 2	39.2 ± 3.9	76 ± 7	627.1 ± 63.0	4 ± 1	56.8 ± 5.7
$(PEA_{1.9}BZA_{0.1})PbBr_4$	9.7 ± 1.0	22 ± 2	39.5 ± 4.0	74 ± 7	546.4 ± 55.1	4 ± 1	53.4 ± 5.4
$(PEA_{1.8}BZA_{0.2})PbBr_4$	10.2 ± 1.0	31 ± 3	41.2 ± 4.1	64 ± 6	519.5 ± 62.0	5 ± 1	52.6 ± 5.3
$(PEA_{1.6}BZA_{0.4})PbBr_4$	10.1 ± 1.0	70 ± 7	42.1 ± 4.2	25 ± 3	508.8 ± 50.9	5 ± 1	45.3 ± 4.5
$(PEA_{1.5}BZA_{0.5})PbBr_4$	10.3 ± 1.0	80 ± 8	51.2 ± 5.2	15 ± 2	507.9 ± 50.8	5 ± 1	43.6 ± 4.4
$(PEABZA)PbBr_4$	8.6 ± 0.9	70 ± 7	29.9 ± 3.0	23 ± 2	294.2 ± 29.4	7 ± 1	33.4 ± 3.3
$(BZA)_2PbBr_4$	4.8 ± 0.5	18 ± 2	20.5 ± 2.1	73 ± 7	537.6 ± 53.8	9 ± 1	66.4 ± 6.6

4. Light yield and quantum yield

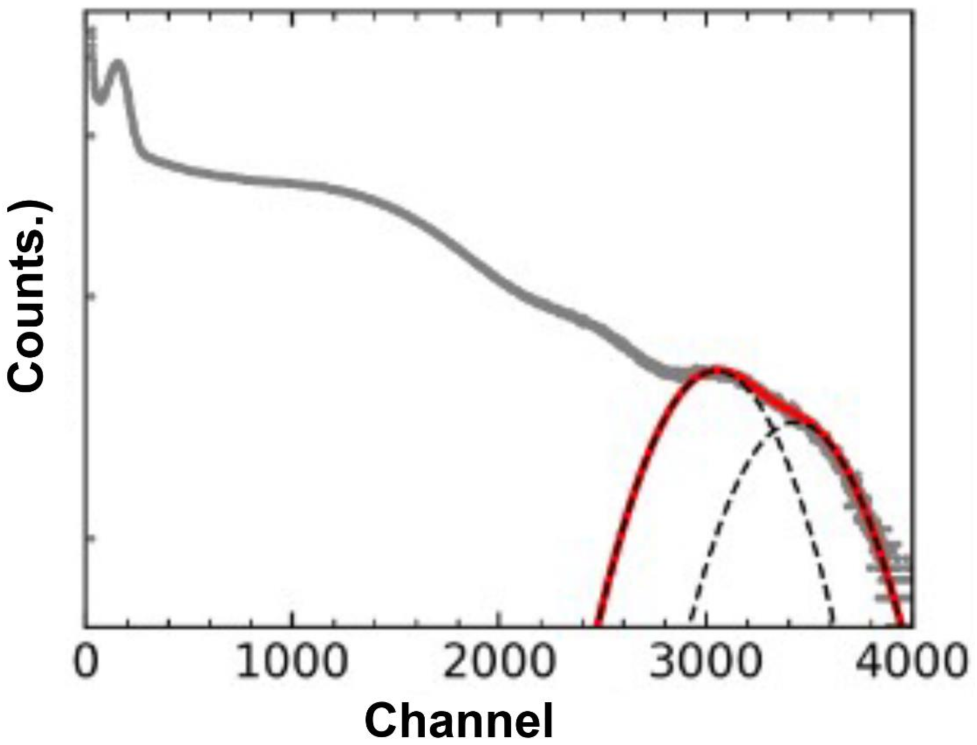

Figure S6. Pulse height spectra (PHS) with 662 keV (${}^{137}Cs$) γ-ray sources for $(BZA)_2PbBr_4$. The dotted gray line indicate the positions of the escape peak and the full-energy peak the red dotted line shows the sum of both peaks.

Table S6. Light Yield obtained from PHS with 59.5 keV (${}^{241}Am$) γ-ray sources at 300 K.

Compounds	Light Yield (ph/keV)
$(PEA)_2PbBr_4$	14.2 ± 1.4
$(PEA_{1.9}BZA_{0.1})PbBr_4$	14.9 ± 1.5
$(PEA_{1.8}BZA_{0.2})PbBr_4$	14.6 ± 1.5
$(PEA_{1.6}BZA_{0.4})PbBr_4$	13.0 ± 1.3
$(PEA_{1.5}BZA_{0.5})PbBr_4$	9.9 ± 1.0
$(PEABZA)PbBr_4$	7.8 ± 0.8
$(BZA)_2PbBr_4$	10.6 ± 1.1

PL quantum yield (QY) of the samples were calculated from the absorbance, refractive index, and PL spectra recorded with integrating sphere of crystals as shown in **Figure S7** and they determined using a modified method from de Mello *et al.* with the following equation.

$$Q_s = Q_r \left(\frac{A_r}{A}\right)\left(\frac{E_s}{E_r}\right)\left(\frac{n_s}{n_r}\right)^2 \quad (S4)$$

Where Q, A, E, and n are the QY, crystal absorbance, intensity of the emitted light from PL, and refractive index, respectively. The subscript "s" and "r" indicate the unknown emitter and $(PEA)_2PbBr_4$ reference sample, from which the properties are already known.[12, 13] The properties obtained from the reference sample are Q_r = 38.2%, n_r = 2.09, and A_r = 0.24 and the details calculation of QY are summarized in **Table S7**.

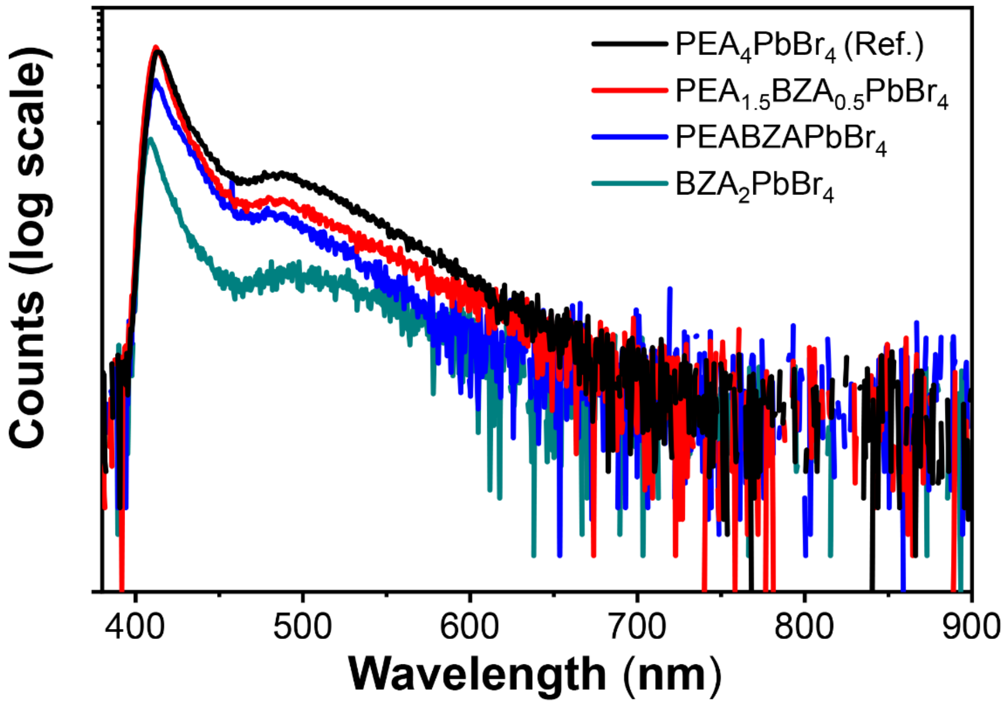

Figure S7. PL spectra recorded with integrating sphere of crystals. $(PEA)_2PbBr_4$, $(PEA_{1.5}BZA_{0.5})PbBr_4$, $(PEABZA)PbBr_4$, and $(BZA)_2PbBr_4$ represent black, red, blue, and dark cyan color, respectively.

Table S7. Calculated quantum yield (QY) for $(PEA)_2PbBr_4$, $(PEA_{1.5}BZA_{0.5})PbBr_4$, $(PEABZA)PbBr_4$, and $(BZA)_2PbBr_4$ samples.

Compounds	Absorbance	Refractive index	PL intensity ratio (E_s/E_r)	QY (%)
$(PEA)_2PbBr_4$	0.24	2.09	1.00	38.2 ± 3.8
$(PEA_{1.5}BZA_{0.5})PbBr_4$	0.49	1.81	0.88	12.3 ± 1.2
$(PEABZA)PbBr_4$	0.32	1.72	0.55	10.7 ± 1.1
$(BZA)_2PbBr_4$	0.35	1.83	0.96	19.3 ± 1.9

5. Negative thermal quenching behavior analysis

For negative thermal quenching behavior analysis, we apply the simple analytical model derived by Shibata[14] to obtain activation energy

$$\|I(T)\| = A_0\,[1+D\cdot\exp(-E_{n1}/k_BT)]\,/\,[\,1+C_1\cdot\exp(-E_{d1}/k_BT)+C_2\cdot\exp(-E_{d2}/k_BT)] \qquad \text{(S5)}$$

where $\|I(T)\|$ is the LY derived from integrated RL intensities under 45 keV X-ray excitation at absolute temperature T, D is the negative thermal quenching coefficient which describes the contribution from thermally excited electrons, C is the thermal quenching coefficient related to non-radiative electron excitation, E_n and E_d are the activation energies for negative thermal quenching and typical thermal quenching and kB is the Boltzmann constant. The fitting parameters are listed in **Table S8**.

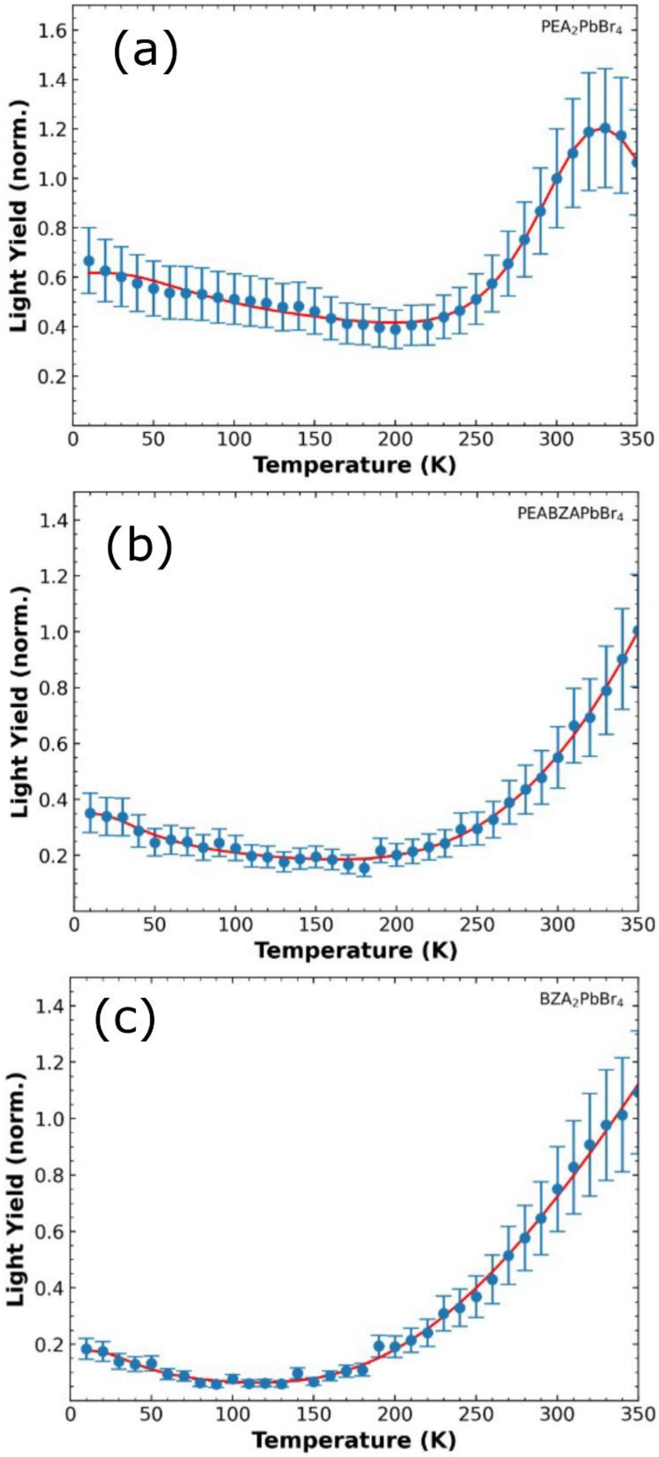

Figure S8. Negative thermal quenching fits using the Shibata model[14] for (a) $(PEA)_2PbBr_4$, (b) $(PEABZA)PbBr_4$, (c) $(BZA)_2PbBr_4$ and the parameters are shown in **Table S8**.

Table S8. Fitting parameters of negative thermal quenching of the radioluminescence using equation S5.

**Compounds**	A_0	D_1	E_{n1} (meV)	C_1	E_{d1} (meV)	C_2	E_{d2} (meV)
$(PEA)_2PbBr_4$	0.61	$2.5x10^4$	242 ± 24	1	12.8 ± 1.3	$2.4x10^7$	475 ± 47
$(PEABZA)PbBr_4$	0.35	$5.8x10^2$	141 ± 14	1.6	7.5 ± 0.7	-	-
$(BZA)_2PbBr_4$	0.17	$5.7x10^2$	91 ± 9	4.8	8.9 ± 0.9	-	-

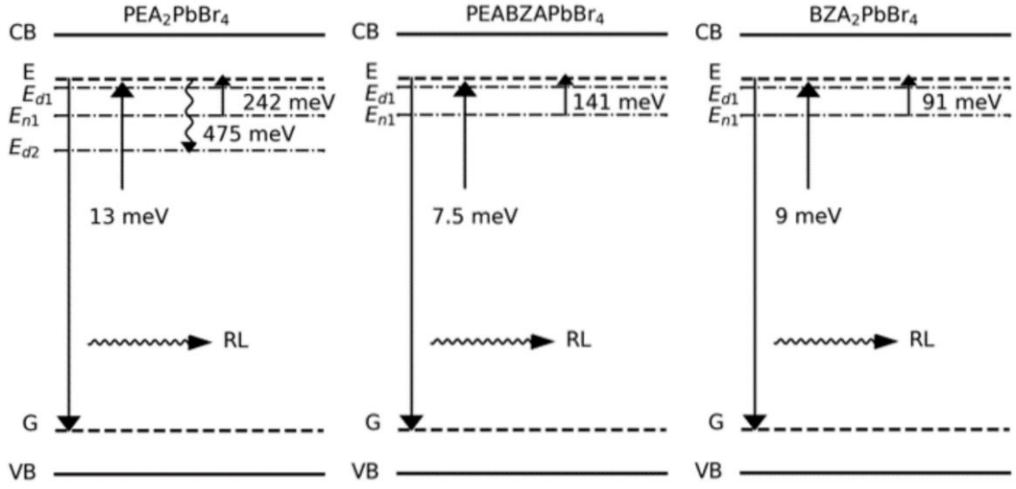

Figure S9. An energy diagram of the model system proposed for the study mechanism of the negative thermal quenching phenomenon for (a) $(PEA)_2PbBr_4$, (b) $(PEABZA)PbBr_4$, (c) $(BZA)_2PbBr_4$.

6. X-ray imaging

Film preparation. The films for the imaging were prepared from a 2 M precursor solution in DMF, with PEABr, BZABr and $PbBr_2$ in stoichiometric amounts. The solution was spin-coated on a glass substrate (with 20 min UV-Ozon treatment) at a speed of 500 rpm for 60 s (acceleration: 100 rpm per s). After the initial 30 s, a heat gun was applied right on top of the film to blow-dry the film with hot air flow for the remainder of the spin-coating time. The film was baked at 100 °C on a hot plate for another 20 min.

X-ray imaging setup. The perovskite on glass films were positioned in front of a LD Didactic 554 800 X-ray apparatus X-ray source (Mo-source, E= 17.5 keV, I= 1 mA, V= 35 keV). Between the X-ray source and the film we placed a Type-18 line pattern card (lead thickness 125 mm). The pattern and the film were put as closed as possible to reduce light scattering. To capture the scintillation of the film we used an Allied Vision Mako U 130B camera, with 1.4 s exposure. Analyzing the acquired images involved calculating the edge spread function and its first derivative, commonly known as the line spread function. Subsequently, we derived the modulation transfer function by obtaining the modulus of the Fourier transform of the line spread function.

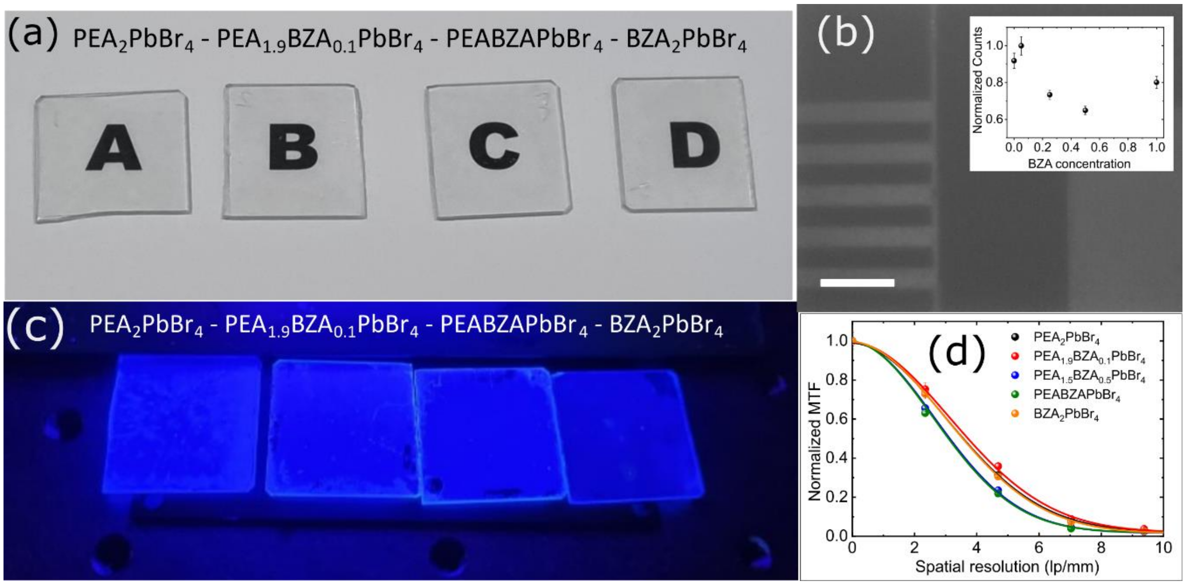

Figure S10. (a) Photographs of the films prepared for X-ray imaging, (b) X-ray imaging of the best ($PEA_{1.9}BZA_{0.1}$)$PbBr_4$ sample (scale bar is 1 mm). The inset shows BZA concentration vs normalized counts, (c) Luminescence (Blue emission) of the samples under UV-light, and (d) Modulation Transfer Function (MTF) of X-ray images obtained from $(PEA)_2PbBr_4$, ($PEA_{1.9}BZA_{0.1}$)$PbBr_4$, ($PEA_{1.5}BZA_{0.5}$)$PbBr_4$, (PEABZA)$PbBr_4$, and $(BZA)_2PbBr_4$ samples with spatial resolution (lp/mm) at 0.2 MTF.

Table S9. Spatial resolution (lp/mm) at 0.2 MTF for $(PEA)_2PbBr_4$, $(PEA_{1.9}BZA_{0.1})PbBr_4$, $(PEA_{1.5}BZA_{0.5})PbBr_4$, $(PEABZA)PbBr_4$, and $(BZA)_2PbBr_4$, Lamellar $CsPbBr_3$ nanocrystals and commercial Gadox (Gd_2O_2S:Tb) layer sample.

Compounds	Spatial resolution (lp/mm) at 0.2 MTF
$(PEA)_2PbBr_4$	5.58
$(PEA_{1.9}BZA_{0.1})PbBr_4$	5.78
$(PEA_{1.5}BZA_{0.5})PbBr_4$	4.87
$(PEABZA)PbBr_4$	4.82
$(BZA)_2PbBr_4$	5.51
$CsPbBr_3$[15]	4.00
Gd_2O_2S:Tb[15]	4.80

REFERENCES

(1) F. Maddalena, M. H. M., D. Kowa, M. E. Witkowski, M. Makowski, Md A. K. Sheikh, S. Mahato, R. Jędrzejewski, W. Drozdowski, C. Dujardin, C. Dang, M. D. Birowosuto. Lattice expansion in Rb-doped hybrid organic–inorganic perovskite crystals resulting in smaller band-gap and higher light-yield scintillators. *Inorg. Chem.* **2023**, *62* (23), 8892-8902. DOI: 10.1021/acs.inorgchem.3c00270.

(2) M. -H. Jung. White-light emission from the structural distortion induced by control of halide composition of two-dimensional perovskites ($(C_6H_5CH_{2n}H_3)_2PbBr_{4-x}cl_x$). *Inorg. Chem.* **2019**, *58*, 6748-6757. DOI: 10.1021/acs.inorgchem.9b00145.

(3) M. D. Birowosuto; D. Cortecchia; W. Drozdowski; K. Brylew; W. Lachmanski; A. Bruno; C. Soci. X-ray scintillation in lead halide perovskite crystals. *Sci. Rep.* **2016**, *6* (1), 37254. DOI: 10.1038/srep37254.

(4) J. T. Randall; M. H. F. Wilkins. Phosphorescence and electron traps I. The study of trap distributions. *Proc. R. Soc. Lond. A* **1945**, *184* (999), 365-389. DOI: 10.1098/rspa.1945.0024.

(5) J. Cao; Z. Guo; S. Zhu; Y. Fu; H. Zhang; Q. Wang; Z. Gu. Preparation of lead-free two-dimensional-layered $(C_8H_{17}NH_3)_2SnBr_4$ perovskite scintillators and their application in X ray imaging. *ACS Appl. Mater. Interfaces* **2020**, *12*, 19797−19804. DOI: 10.1021/acsami.0c02116.

(6) R. J. Elliott. Theory of the effect of spin-orbit coupling on magnetic resonance in some semiconductors. *Phys. Rev.* **1954**, *96* (2), 266-279. DOI: 10.1103/PhysRev.96.266.

(7) P. Avouris; T. N. Morgan. A tunneling model for the decay of luminescence in inorganic phosphors: Thecase of Zn2SiO4:Mn. *J. Chem. Phys.* **1981**, *74*, 4347-4355. DOI: 10.1063/1.441677.

(8) K. Brylew, W. D., A. J. Wojtowicz, K. Kamada and A. Yoshikawa. Studies of low temperature thermoluminescence of GAGG:Ce and LuAG:Pr scintillator crystals using the Tmax-Tstop method. *J. Lumin.* **2014**, *154*, 452-457. DOI: 10.1016/j.jlumin.2014.05.035.

(9) W. Drozdowski, K. B., M.E. Witkowski, A.J. Wojtowicz, P. Solarz, K. Kamada and A. Yoshikawa. Studies of light yield as a function of temperature and low temperature thermoluminescence of $Gd_3Al_2Ga_3O_{12}$:Ce scintillator crystals. *Opt. Mater.* **2014**, *36* (10). DOI: 10.1016/j.optmat.2013.12.044.

(10) W. Drozdowski, K. B., M. Malinowski and S. Turczynski. Scintillation properties ofμPD-grown $Y_4Al_2O_9$:Pr(YAM:Pr) crystals. *J. Alloys Compd.* **2015**, *632*, 816-821. DOI: 10.1016/j.jallcom.2015.01.274.

(11) M. Makowski, M. E. W., W. Drozdowski, G. Zhang, J. Li, Y. Wu. Scintillation properties of $(Ga_xAl_{1-x})_2O_3$ ceramics. *Radiat. Phys. Chem.* **2023**, *208* (110896). DOI: 10.1016/j.radphyschem.2023.110896.
(12) W. Ye; Z. Yong; M. Go; D. Kowal; F. maddalena; L. Tjahjana; W. Hong; Arramel; C. Dujardin; M. D. Birowosuto; et al. The nanoplasmonic purcell effect in ultrafast and high-light-yield perovskite scintillators. *Adv. Mater.* **2023**. DOI: 10.48550/arXiv.2309.06320.
(13) J. C. De Mello; H. F. Wittmann; Friend, R. H. An improved experimental determination of external photoluminescence quantum efficiency. *Adv. Mater.* **1997**, *9*, 230-232. DOI: 10.1002/adma.19970090308.
(14) Shibata, H. Negative thermal quenching curves in photoluminescence of solids. *Jpn. J. Appl. Phys.* **1998**, *37*, 550-553. DOI: 10.1143/JJAP.37.550.
(15) F. Maddalena; M. E. Witkowski; M. Makowski; A. Bachiri; B. Mahler; Y.-C. Wong; C. Y. E. Chua; J. X. Lee; W. Drozdowski; S. V. Springham; et al. Stable and bright commercial $CsPbBr_3$ quantum dot-resin layers for apparent X-ray imaging screen. *ACS Applied Materials & Interfaces* **2021**, *13* (49), 59450-59459. DOI: 10.1021/acsami.1c16171.
